# Selecting Invalid Instruments to Improve Mendelian Randomization with Two-Sample Summary Data

**DOI:** 10.1214/23-AOAS1856

**Published:** 2024-04-05

**Authors:** Ashish Patel, Francis J. DiTraglia, Verena Zuber, Stephen Burgess

**Affiliations:** 1 MRC Biostatistics Unit, University of Cambridge; 2 Department of Economics, University of Oxford; 3 Department of Biostatistics and Epidemiology, Imperial College London; 4 Cardiovascular Epidemiology Unit, University of Cambridge

**Keywords:** Mendelian randomization, focused information criterion, post-selection inference

## Abstract

Mendelian randomization (MR) is a widely-used method to estimate the causal relationship between a risk factor and disease. A fundamental part of any MR analysis is to choose appropriate genetic variants as instrumental variables. Genome-wide association studies often reveal that hundreds of genetic variants may be robustly associated with a risk factor, but in some situations investigators may have greater confidence in the instrument validity of only a smaller subset of variants. Nevertheless, the use of additional instruments may be optimal from the perspective of mean squared error even if they are slightly invalid; a small bias in estimation may be a price worth paying for a larger reduction in variance. For this purpose, we consider a method for “focused” instrument selection whereby genetic variants are selected to minimise the estimated asymptotic mean squared error of causal effect estimates. In a setting of many weak and locally invalid instruments, we propose a novel strategy to construct confidence intervals for post-selection focused estimators that guards against the worst case loss in asymptotic coverage. In empirical applications to: (i) validate lipid drug targets; and (ii) investigate vitamin D effects on a wide range of outcomes, our findings suggest that the optimal selection of instruments does not involve only a small number of biologically-justified instruments, but also many potentially invalid instruments.

## 1 Introduction

Mendelian randomization (MR) uses genetic variants as instrumental variables to estimate the causal effect of a risk factor on an outcome in the presence of unobserved confounding. By Mendel’s second law, genetic variants sort independently of other traits. Thus, genetic variants, which are fixed at conception, can provide a source of exogenous variation in a risk factor of interest, allowing analyses that are less vulnerable to reverse causality and confounding (Davey Smith and Ebrahim, 2003).

Large-scale consortia genome-wide association studies (meta-GWASs) have identified large numbers of genetic variants that are robustly associated with a wide range of traits. Due in part to privacy issues, often only summary statistics of these genetic associations are made publicly available. Since these results are easily accessible, MR investigations increasingly rely on inferential methods that require only two-sample summary data (Burgess et al., 2015). In such applications, genetic variant associations with the risk factor are obtained from a representative but non-overlapping sample used to measure genetic variant associations with the outcome.

**Fig 1:**
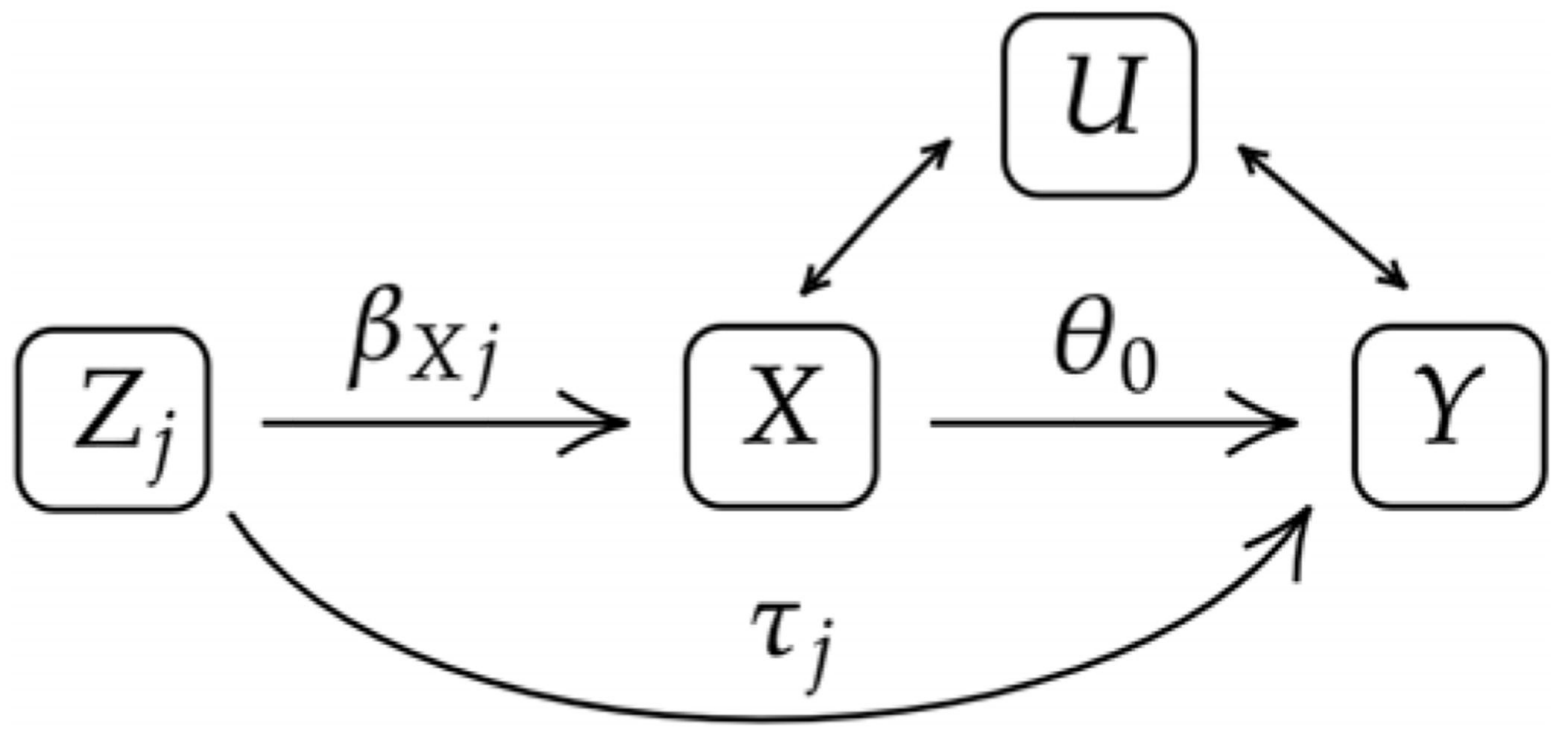
The effect of genetic variant Z_j_ on the risk factor X and outcome Y, where U is an unobserved confounder.

As in usual instrumental variable analyses, identifying causal effects through MR requires some key assumptions. For a genetic variant to be a valid instrument, it must be associated with the risk factor; *β_X_j__* ≠ 0 in Figure 1 (relevance). Second, the variant must be uncorrelated with unobserved confounders. Third, any effect that the variant has on the outcome must be mediated by its effect on the risk factor; *τ_j_* = 0 in Figure 1 (exclusion).

Violations of the exclusion condition are common in MR studies (Lawlor et al., 2008), due in part to the widespread phenomenon of pleiotropy where a single genetic variant may influence several traits (Solovieff et al., 2013; Hemani, Bowden and Davey Smith, 2018). While the biological mechanism of certain genetic effects may be well understood (for example, when using protein risk factors for drug target validation; see Schmidt et al., 2020), it is generally difficult to rule out the possibility that many genetic variants have a direct effect on the outcome, and thus violate the exclusion restriction (Verbanck et al., 2018).

In some MR applications, investigators may have more confidence in the validity of a particular subset of all candidate instruments. In particular, the causal mechanisms linking specific genes to risk factors may be known, which may better justify the use of genetic variants from those genes as instruments. Examples of MR studies that have prioritised the use of variants from biologically-plausible genes include investigations into the effects of alcohol consumption (Millwood et al., 2019), C-reactive protein level (Swerdlow et al., 2016), smoking behaviour (Lassi et al., 2016), vitamin D supplementation (Mokry et al., 2015), and perturbing drug targets (Gill et al., 2021).

Along with biologically-justified instruments, investigators could consider using additional variants that are plausibly valid instruments in order to improve the precision of an analysis. Even if these additional instruments are slightly invalid, their use may provide slightly biased but more precise estimates that are optimal from the perspective of mean squared error.

Hence, to guide this instrument choice, we propose a method for “focused” instrument selection whereby genetic variants are selected to minimise the estimated asymptotic mean squared error of causal effect estimates. The strategy allows a *tiering* in the assumptions on instrument validity, and prioritises the evidence suggested by biologically-justified instruments.

We work with the popular two-sample summary data design, and in a setting of many weak and locally invalid instruments. In this local mis-specification setting, the collective direct instrument effects on the outcome are decreasing with the sample size at a rate that ensures a meaningful bias-variance trade off, and thus enables a mean squared error comparison in finite samples.

The theoretical contribution of our work has two elements. First, we extend DiTraglia (2016)’s results to allow for many weak instruments. We consider an asymptotic framework in which the number of instruments can grow at the same rate as the sample size, as long as their collective effects on the risk factor are bounded (Zhao et al., 2020). Second, we consider the problem of post-selection inference for focused estimators which is particularly challenging because we do not have consistent model selection; the uncertainty in model selection directly impacts the asymptotic distribution of focused estimators.

Given that focused use of additional instruments may lead to improved estimation compared with using only a smaller set of biologically-justified instruments, it is natural to consider whether a similar advantage may hold for inference. One desirable property for confidence intervals is that they are *uniformly valid*; that is, they achieve nominal coverage asymptotically over the space of potential direct instrument effects on the outcome. Unfortunately, the impossibility results discussed by Leeb and Pötscher (2005) suggest we cannot construct uniformly valid confidence intervals for focused estimators which will exactly achieve nominal coverage, and therefore such intervals will generally be conservative. For example, in simulation we find that the “2-step” uniformly valid confidence intervals of DiTraglia (2016) can be over 30% longer than standard confidence intervals based on using only a core set of valid instruments.

In the same way that focused estimation is willing to trade a small bias in estimation for a larger reduction in variance, we develop a strategy for constructing “focused” confidence intervals that accepts a small loss in coverage probability over a subspace of direct instrument effects on the outcome in exchange for shorter confidence intervals. Our focused confidence intervals account for uncertainty in model selection, and they achieve nominal coverage for plausible values of direct instrument effects, while providing a statistical guarantee on the worst case size distortion away from those values.

Compared with using only a core set of biologically-justified instruments, our simulation evidence illustrates how focused estimators and confidence intervals may provide improved estimation and inference when additional instruments are valid or slightly invalid, while *buying insurance* against poor performance when additional instruments are very invalid.

The utility of our methods is demonstrated in two empirical applications. The first considers genetic validation of lipid drug targets when investigators are uncertain about defining the width of a *cis*-gene window from which to select instruments. The second application investigates the genetically predicted effect of vitamin D supplementation on a range of outcomes when investigators want to prioritise the use of genetic variants from specific genes through biological considerations.

We use the following notation and abbreviations: $\underset{\to }{P}$ ‘converges in probability to’; $\underset{\to }{D}$ ‘converges in distribution to’; $\underset{\sim }{a}$ ‘is asymptotically distributed as’. For any sequences *a_n_* and *b_n_*, if *a_n_* = *O*(*b_n_*), then there exists a positive constant *M* and a positive integer *N* such that for all *n* ≥ *N*, *b_n_* > 0 and |*a_n_*| ≤ *Mb_n_*. If *a_n_* = *o*(*b_n_*), then |*a_n_*|/*b_n_* → 0 as *n* → ∞. Also, if *a_n_* = Θ(*b_n_*), then there exist positive constants *M*_1_ and *M*_2_, *M*_1_ ≤ *M*_2_ < ∞, and a positive integer *N* such that *M*_1_*b_n_* ≤ *a_n_* ≤ *M*_2_*b_n_* for all *n* ≥ *N*. The proofs of theoretical results are given in Supplementary Material (Patel et al., 2024), and R code to perform our empirical investigation is available on GitHub at github.com/ash-res/focused-MR/.

## 2 Model and assumptions

### 2.1 Two-sample summary data

We first outline our assumptions on genetic association summary data, which are motivated through a simple linear model with invalid instruments and homoscedastic errors.

Let *Z* = (*Z*_1_,…, *Z_p_*)′ denote a *p*-vector of uncorrelated genetic variants. The parameter of interest is the causal effect *θ*_0_ of the risk factor *X* on the outcome *Y*, which is described by

(1) $$Y=\omega_{Y}+X\theta_{0}+Z^{\prime }\tau +U_{Y}$$

(2) $$X=\omega_{X}+Z^{\prime }\beta_{X}+U_{X},$$

where $E\left[U_{Y}|Z\right]=0,E\left[U_{X}|Z\right]=0,E\left[U_{Y}^{2}|Z\right]=\sigma_{U_{Y}}^{2},E\left[U_{X}^{2}|Z\right]=\sigma_{U_{X}}^{2},\beta_{X}={\left(\beta_{X_{1}},\ldots ,\beta_{X_{p}}\right)}^{\prime }$, and (*ω_X_, ω_Y_, β_X_, τ, θ*_0_) are unknown parameters. If *τ* is non-zero, then at least one genetic variant fails the exclusion restriction and directly affects the outcome.

Substituting (2) into (1), we have *Y* = (*ω_Y_* + *ω_X_θ*_0_) + *Z′*(*β_X_θ*_0_ + *τ*) + (*U_Y_* + *U_X_θ*_0_). Thus, *Cov*(*Z, Y*) = *Var*(*Z*)(*β_X_θ*_0_ + *τ*), which leads to a model

(3) $$\beta_{Y}=\beta_{X}\theta_{0}+\tau ,$$

where *β_Y_* = (*β*_*Y*_1__,…, *β_Y_p__*)′ is the *p*-vector of coefficients from a population regression of *Y* on *Z*. We aim to estimate the model in (3) using two-sample summary data on genetic associations.

#### Assumption 1 (two-sample summary data)

*For each variant j, we observe genetic associations* ${\hat{\beta }}_{X_{j}}$ *and* ${\hat{\beta }}_{Y_{j}}$, *which satisfy* ${\hat{\beta }}_{X_{j}}\sim N\left(\beta_{X_{j}},\sigma_{X_{j}}^{2}\right)$ *and* ${\hat{\beta }}_{Y_{j}}\sim N\left(\beta_{Y_{j}},\sigma_{Y_{j}}^{2}\right)$*, where* $\sigma_{X_{j}}^{2}=\Theta \left(1/n_{X}\right)$ *and* $\sigma_{Y_{j}}^{2}=\Theta \left(1/n_{Y}\right)$ *are assumed to be known. Moreover, the set of* 2*p genetic associations* ${\left\{{\hat{\beta }}_{X_{j}},{\hat{\beta }}_{Y_{j}}\right\}}_{j=1}^{p}$ *are mutually uncorrelated, and n_X_/n_Y_* → *c, as n* := (*n_X_, n_Y_*) → ∞ *for some constant* 0 < *c* < ∞.

Assumption 1 is taken from Zhao et al. (2020) and states a normal approximation of estimated genetic associations which is typically justified by large random sampling expected in genetic association studies. Specifically, for each variant *j*, we have access to estimates and standard errors from univariable *X* on *Z_j_* linear regressions from an *n_X_*-sized sample, and from a non-overlapping *n_Y_*-sized sample, we observe measured associations from univariable *Y* on *Z_j_* linear regressions. Both random samples are drawn from the joint distribution of (*Y, X, Z*).

The assumption that the population standard deviations ${\left\{\sigma_{X_{j}},\sigma_{Y_{j}}\right\}}_{j=1}^{p}$ are known is common in MR, and a formal justification for this is given by Ye, Shao and Kang (2021). To simplify notation, the dependence of the standard errors on the sample size is not made explicit, but they are assumed to decrease at the usual parametric rate.

### 2.2 Core instruments

From (3), the parameter of interest *θ*_0_ is not identified unless there are some restrictions on *τ* = (*τ*_1_,…, *τ_p_*)′. When genetic variants from several gene regions are used to instrument the risk factor, a popular identification strategy is to assume that *τ* is a mean zero random effect; see, for example, Zhao et al. (2020). Another commonly-used assumption is that most genetic variants are valid instruments; *τ_j_* = 0 for *j* ∈ *S_M_*, where *S_M_* is some unknown set of variants such that *p*^−1^|*S_M_*| > 0.5. In this setting the median-based estimators of Bowden et al. (2016) are consistent. Based on a variation of these assumptions, many summary data MR methods have proposed ways to obtain unbiased estimates of *θ*_0_; a recent review is given in Sanderson et al. (2022).

In this work we do not focus on a novel identification strategy, but instead only the simple instrument selection choice for investigators when they believe a core set of genetic variants *S*_0_ consists of only valid instruments, but they are less confident on the validity of any additional candidate instruments.

#### Assumption 2 (core instruments)

*For some known set of instruments S*_0_ *such that* 1 ≤ |*S*_0_| < *p, and where* |*S*_0_| *grows proportionately with p, we have τ_j_* = 0 *for all j* ∈ *S*_0_.

Assumption 2 allows us to measure the bias which may result from the inclusion of additional instruments. In some MR studies, there is good reason for believing that a smaller subset of all available genetic variants are more likely to be valid instruments. We mention two examples that we explore in more detail in Section 6.

#### Example 1 (choosing *cis* windows in drug target MR)

*In drug target MR studies, only those genetic variants from a single gene region that encodes the protein target of a drug are used to instrument the risk factor. Such studies have gained popularity for providing supporting genetic evidence to validate drug targets and to study side effects (Gill et al., 2021). A key decision in drug target MR is to choose the width of a “cis window” which dictates a gene region from which to select instruments. Tools such as GeneCards (Stelzer et al., 2016) offer practical guidance on defining appropriate gene regions, but since there are often relatively few uncorrelated genetic signals from narrow cis windows, researchers often resort to widening the cis window in order to boost power through the use of a larger number of instruments. This leaves the possibility of a type of publication bias where researchers may report findings only from a cis window which gives their preferred result. We may be less confident on the instrument validity of additional genetic variants that are included only from widening a cis window.*

#### Example 2 (estimating vitamin D effects)

*GWASs have identified strong genetic associations with vitamin D in biologically plausible genes; GC, DHCR7, CYP2R1, and CYP24A1. Each of these genes is known influence vitamin D level through different mechanisms. In order to investigate the effect of vitamin D supplementation on a range of traits and diseases, MR studies have often used genetic variants located in neighborhoods of those genes to instrument vitamin D as they are considered more likely to satisfy the exclusion restriction than other genome-wide significant variants (Mokry et al., 2015; Revez et al., 2020). Genetic variants from other gene regions may be strongly associated with vitamin D, but they may be more likely to have direct effects on the outcome through their effects on traits other than vitamin D.*

### 2.3 Many weak variant associations

In typical applications, we may expect many genetic variants to have weak effects on the risk factor. This can cause difficulties for identifying and estimating the causal effect. We study a setting of many weak instruments where the number of genetic variants is permitted to grow at the same rate as the sample size, but their collective explanatory power is bounded.

#### Assumption 3 (many weak instruments)

*Let β*_*X*,0_ *denote a* |*S*_0_|-*vector with its elements given by β_X_j__ for j* ∈ *S*_0_. *Then,* ||*β_X_*||_2_ = *O*(1), ||*β_X_*||_3_/||*β*_*X*,0_||_2_ → 0, *and* $p/n{\left\|\beta_{X,0}\right\|}_{2}^{2}=O\left(1\right)$ *as n, p* → ∞.

Given (3) and Assumptions 2 and 3, the causal effect is point identified. Assumption 3 is similar to Zhao et al. (2020), and it implies that all variants are relevant instruments, but the explanatory power of any individual variant is decreasing as *p* → ∞. Moreover, the skewness of *β_X_* is restricted which rules out very sparse variant effects settings. Finally, the rate restriction $p/n{\left\|\beta_{X,0}\right\|}_{2}^{2}=O\left(1\right)$ as *n, p* → ∞ ensures asymptotic normality in estimation, and the condition is plausible given the large sample sizes of typical GWASs.

### 2.4 Additional locally invalid instruments

To meaningfully study a bias-variance trade off from including additional instruments we need to ensure that the bias and variance terms have the same order of magnitude. Following DiTraglia (2016)’s framework of *locally invalid* instruments, we work with local mis-specification in which the direct effects *τ* are collectively “local-to-zero”, and decrease at the same rate as the sampling errors with *n*. This is not a substantive biological assumption, but rather a technical device that allows us to study the instrument selection problem from a mean squared error perspective.

#### Assumption 4 (locally invalid instruments)

${\left\|\tau \right\|}_{2}^{2}=O\left(1/n\right)$ *as n, p* → ∞.

Under the rate restriction in Assumption 4, we can consistently estimate *θ*_0_ using any set of instruments, but making valid inferences using invalid instruments will require us to account for an asymptotic bias. The rate restriction is slightly different to DiTraglia (2016) in that we limit only the collective direct effects over all variants.

Assumption 4 is otherwise quite general. For example, the direct effects *τ* may be correlated with *β_X_*, and thus may violate the so-called InSIDE (Instrument Strength Independent of Direct Effect) assumption which some existing MR methods that make use of invalid instruments rely on (Bowden, Smith and Burgess, 2015).

## 3 Focused instrument selection and estimation

Using only the core set of instruments *S*_0_ should lead to asymptotically unbiased estimation of *θ*_0_, and this forms a basis from which to measure the potential bias from including additional instruments. For ease of exposition we focus on the simple choice between using either: (i) the “Core” estimator that chooses only the set of core instruments *S*_0_; or (ii) the “Full” estimator that uses the full set of *p* instruments. The inclusion of *S*, the set of the additional *p* – |*S*_0_| genetic variants, may result in improved estimation if there is a large reduction in variance, and at most only a small increase in bias. Thus we use a “focused” instrument selection strategy (DiTraglia, 2016) where the Full estimator is selected only if it has a lower estimated asymptotic mean squared error than the Core estimator.

The results presented here can be easily extended for more fine-tuned instrument selection at the expense of extra notation; instead of simply choosing between the Core and Full estimators, we could also select from subsets of the additional instruments. Such subsets could be data-driven, chosen on biological reasons, and have overlapping variants. For example, in our empirical application to study vitamin D effects, we chose from all possible subsets of 3 partitions of the additional instruments, where the partitions were formed by k-means clustering on the Wald ratio estimate of each variant, ${\hat{\beta }}_{Y_{j}}/{\hat{\beta }}_{X_{j}},j\in S$.

We consider limited information maximum likelihood (LIML) estimation of *θ*_0_. The Core and Full estimators, ${\hat{\theta }}_{C}$ and ${\hat{\theta }}_{F}$, are given by

$${\hat{\theta }}_{C}=\arg\underset{\theta }{\min}\sum_{j\in S_{0}}\frac{{\left({\hat{\beta }}_{Y_{j}}-{\hat{\beta }}_{X_{j}}\theta \right)}^{2}}{\sigma_{Y_{j}}^{2}+\sigma_{X_{j}}^{2}\theta^{2}}\;\text{and}\;{\hat{\theta }}_{F}=\arg\underset{\theta }{\min}\sum_{j\in S_{0}\cup S}\frac{{\left({\hat{\beta }}_{Y_{j}}-{\hat{\beta }}_{X_{j}}\theta \right)}^{2}}{\sigma_{Y_{j}}^{2}+\sigma_{X_{j}}^{2}\theta^{2}}.$$

Under Assumptions 1-3, Theorem 3.1 of Zhao et al. (2020) shows that the asymptotic distribution of ${\hat{\theta }}_{C}$ is

$$\Delta_{C}^{-1/2}\left({\hat{\theta }}_{C}-\theta_{0}\right)\overset{D}{\to }N\left(0,1\right),$$

as *n, p* → ∞, where $\Delta_{C}=\eta_{C}^{-2}\left(\eta_{C}+\zeta_{C}\right),\eta_{C}=\sum_{j\in S_{0}}\Omega_{j}^{-1}\beta_{X_{j}}^{2},\zeta_{C}=\sum_{j\in S_{0}}\Omega_{j}^{-2}\sigma_{X_{j}}^{2}\sigma_{Y_{j}}^{2}$, and $\Omega_{j}=\sigma_{Y_{j}}^{2}+\theta_{0}^{2}\sigma_{X_{j}}^{2}$. Using similar arguments, we can derive the asymptotic distribution of the Full estimator.

### Theorem 1 (Full estimator)

*Under Assumptions 1-4, and the Full estimator is consistent, and its asymptotic distribution is*

$$\Delta_{F}^{-1/2}\left({\hat{\theta }}_{F}-\theta_{0}-b\right)\overset{D}{\to }N\left(0,1\right),$$

*as n, p* → ∞, *where* $\Delta_{F}={\left(\eta_{C}+\eta_{S}\right)}^{-2}\left(\eta_{C}+\eta_{S}+\zeta_{C}+\zeta_{S}\right),b={\left(\eta_{C}+\eta_{S}\right)}^{-1}b_{S},b_{S}=\sum_{j\in S}\Omega_{j}^{-1}\beta_{X_{j}}\tau_{j},\eta_{S}=\sum_{j\in S}\Omega_{j}^{-1}\beta_{X_{j}}^{2}$, *and* $\zeta_{S}=\sum_{j\in S}\Omega_{j}^{-2}\sigma_{X_{j}}^{2}\sigma_{Y_{j}}^{2}$.

The variance terms *η_C_* and *η_S_* are of order $O\left(n{\left\|\beta_{X}\right\|}_{2}^{2}\right)$, and the terms *ς_C_* and *ς_S_* are of order *O*(*p*). Therefore, (*η_C_* + *η_S_*)^−1^ would be the asymptotic variance of ${\hat{\theta }}_{F}$ in a fixed *p* setting with strong instruments, and (*η_C_* + *η_S_*)^−2^(*ς_C_* + *ς_S_*) is an additional variance component to account for the extra uncertainty due to many weak instruments (Zhao et al., 2020). This is particularly important for our instrument selection problem since under-estimated variances based on ‘fixed *p*’ asymptotics could cause a mean squared error-based selection criteria to falsely recommend the inclusion of additional instruments. These two variance components are of the same order of magnitude when $p/n{\left\|\beta_{X}\right\|}_{2}^{2}=\Theta \left(1\right)$.

As discussed by Newey and Windmeijer (2009), despite the knife-edge condition required to balance these variance components, it may be advisable to use the weak instrument variance correction (*η_C_* + *η_S_*)^−2^(*ς_C_* + *ς_S_*) in general scenarios when *n* is considerably larger than *p*, and when instruments are strong. For example, the simulation study of Davies et al. (2015, pp. 457-9), which mimicked an MR design, showed that standard errors that did not correct for many weak instrument effects led to inflated type I error rates even for the case where *n* = 3000 and *p* = 9.

We also note that compared with the model of Zhao et al. (2020), here we consider *τ* to be a fixed effect rather than a random variable; this direct variant effect on the outcome induces a bias in estimation rather than an increase in variance. The asymptotic variance of ${\hat{\theta }}_{F}$ is of order $O\left(1/n{\left\|\beta_{X}\right\|}_{2}^{2}\right)+O\left(p/n^{2}{\left\|\beta_{X}\right\|}_{2}^{4}\right)$, and its asymptotic bias is of order $O\left(1/\sqrt{n}{\left\|\beta_{X}\right\|}_{2}\right)$. Thus, there is a meaningful bias-variance trade off if $p/n{\left\|\beta_{X}\right\|}_{2}^{2}=O\left(1\right)$ since the square of the asymptotic bias is of the same order of magnitude as the asymptotic variance.

To carry out focused instrument selection, we need to estimate and compare the asymptotic mean squared error (AMSE) of ${\hat{\theta }}_{C}$ and ${\hat{\theta }}_{F}$. The Core estimator is asymptotically unbiased, and it is straightforward to consistently estimate its asymptotic variance Δ_*C*_. Under local mis-specification, the asymptotic bias *b* of the Full estimator cannot be consistently estimated. However, we can use ${\hat{\theta }}_{C}$ to construct an asymptotically unbiased estimate of *b*.

Let ${\hat{\eta }}_{C}=\sum_{j\in S_{0}}{\hat{\Omega }}_{j}^{-1}\left({\hat{\beta }}_{X_{j}}^{2}-\sigma_{X_{j}}^{2}\right),{\hat{\eta }}_{S}=\sum_{j\in S}{\hat{\Omega }}_{j}^{-1}\left({\hat{\beta }}_{X_{j}}^{2}-\sigma_{X_{j}}^{2}\right)$, and ${\hat{\Omega }}_{j}=\sigma_{Y_{j}}^{2}+{\hat{\theta }}_{C}^{2}\sigma_{X_{j}}^{2}$. Then, our estimator of *b* is $\hat{b}={\left({\hat{\eta }}_{C}+{\hat{\eta }}_{S}\right)}^{-1}{\hat{b}}_{S}$, where ${\hat{b}}_{S}=\sum_{j\in S}{\hat{\Omega }}_{j}^{-1}{\hat{\beta }}_{X_{j}}{\hat{\beta }}_{Y_{j}}-{\hat{\theta }}_{C}\sum_{j\in S}{\hat{\Omega }}_{j}^{-1}\left({\hat{\beta }}_{X_{j}}^{2}-\sigma_{X_{j}}^{2}\right)$.

### Theorem 2 (Asymptotic bias estimator)

*Under Assumptions 1-4, the asymptotic distribution of* $\hat{b}$ *is*

$$\Delta_{B}^{-1/2}\left(\hat{b}-b\right)\overset{D}{\to }N\left(0,1\right),$$

*as n, p* → ∞, *where* $\Delta_{B}={\left(\eta_{C}+\eta_{S}\right)}^{-2}\left[\eta_{S}+\zeta_{S}+\xi_{S}+\eta_{C}^{-2}\eta_{S}^{2}\left(\eta_{C}+\zeta_{C}\right)\right]$ *and* $\xi_{S}=2\theta_{0}^{2}\sum_{j\in S}\Omega_{j}^{-2}\sigma_{X_{j}}^{4}$.

The standard error of the estimate $\hat{b}$ can be up to the same order of magnitude $O_{P}\left(1/\sqrt{n}{\left\|\beta_{X}\right\|}_{2}\right)$ as the true bias *b*, and hence the asymptotic variance of $\hat{b}$ does not decrease fast enough for consistency as *n, p* → ∞. However, Theorem 2 shows that $\hat{b}$ is an asymptotically unbiased estimator. Similar to before, we can write the asymptotic variance Δ_*B*_ as the sum of two terms: ${\left(\eta_{C}+\eta_{S}\right)}^{-2}\eta_{S}\left(1+\eta_{C}^{-1}\eta_{S}\right)$ and ${\left(\eta_{C}+\eta_{S}\right)}^{-2}\left(\zeta_{S}+\xi_{S}+\eta_{C}^{-2}\eta_{S}^{2}\zeta_{C}\right)$. The first term is the asymptotic variance of $\hat{b}$ in a fixed *p* setting with strong instruments, and therefore the second term represents the extra uncertainty in estimation due to many weak instruments.

In order to estimate the AMSE of ${\hat{\theta }}_{C}$ and ${\hat{\theta }}_{F}$, we need to construct consistent estimators ${\hat{\Delta }}_{C}={\hat{\eta }}_{C}^{-2}\left({\hat{\eta }}_{C}+{\hat{\varsigma }}_{C}\right)$ for $\Delta_{C},{\hat{\Delta }}_{F}={\left({\hat{\eta }}_{C}+{\hat{\eta }}_{S}\right)}^{-2}\left({\hat{\eta }}_{C}+{\hat{\eta }}_{S}+{\hat{\varsigma }}_{C}+\varsigma_{S}\right)$ for Δ_F_, and ${\hat{\Delta }}_{B}={\left({\hat{\eta }}_{C}+{\hat{\eta }}_{S}\right)}^{-2}\left[{\hat{\eta }}_{S}+{\hat{\varsigma }}_{S}+{\hat{\xi }}_{S}+{\hat{\eta }}_{C}^{-2}{\hat{\eta }}_{S}^{2}\left({\hat{\eta }}_{C}+{\hat{\varsigma }}_{C}\right)\right]$ for Δ_*B*_ where ${\hat{\varsigma }}_{C}=\sum_{j\in S_{0}}{\hat{\Omega }}_{j}^{-2}\sigma_{X_{j}}^{2}\sigma_{Y_{j}}^{2},{\hat{\varsigma }}_{S}=\sum_{j\in S}{\hat{\Omega }}_{j}^{-2}\sigma_{X_{j}}^{2}\sigma_{Y_{j}}^{2}$, and ${\hat{\xi }}_{S}=2{\hat{\theta }}_{C}^{2}\sum_{j\in S}{\hat{\Omega }}_{j}^{-2}\sigma_{X_{j}}^{4}$.

Since ${\hat{\theta }}_{C}$ is asymptotically unbiased, a consistent estimator for its AMSE is ${\hat{\Delta }}_{C}$. Whereas for ${\hat{\theta }}_{F}$, an asymptotically unbiased estimator of its AMSE is $\left({\hat{b}}^{2}-{\hat{\Delta }}_{B}\right)+{\hat{\Delta }}_{F}$. Following DiTraglia (2016), since the square of the asymptotic bias cannot be negative, we use $\max\left(0,{\hat{b}}^{2}-{\hat{\Delta }}_{B}\right)$ instead of ${\hat{b}}^{2}-{\hat{\Delta }}_{B}$ when estimating the AMSE of ${\hat{\theta }}_{F}$.

Define $\hat{W}=\max\left({\hat{b}}^{2}-{\hat{\Delta }}_{B},0\right)+{\hat{\Delta }}_{F}-{\hat{\Delta }}_{C}$ as the estimated AMSE of ${\hat{\theta }}_{F}$ minus the estimated AMSE of ${\hat{\theta }}_{C}$. Therefore, the selection event that we select the Full estimator is given by $\left\{\hat{W}\leq 0\right\}$. The “Focused” estimator is then given by

$$\hat{\theta }=I\left\{\hat{W}\leq 0\right\}{\hat{\theta }}_{F}+\left(1-I\left\{\hat{W}\leq 0\right\}\right){\hat{\theta }}_{C}.$$

Since both ${\hat{\theta }}_{F}$ and ${\hat{\theta }}_{C}$ are consistent estimators of *θ*_0_, so is the Focused estimator $\hat{\theta }$.

As noted by a reviewer, it is also possible to use Theorem 2 to construct a valid test of *H*_0_ : *b* = 0. Such tests could potentially be used to inform an alternative instrument selection strategy based on pre-testing for bias; see Supplementary Material for further discussion (Patel et al., 2024).

## 4 Post-selection inference

In this section we discuss the problem of constructing confidence intervals for the Focused estimator $\hat{\theta }$, which is non-standard because model selection is based on an AMSE criterion that is not consistently estimated.

We start by deriving the asymptotic distribution of $\hat{\theta }$, and discuss why naive confidence intervals that ignore sampling uncertainty in instrument selection are likely to perform poorly. We then note the relative merits of two related inference procedures proposed in DiTraglia (2016), before introducing a new “Focused” approach that combines their strengths. Just as the Focused estimator aims to achieve a good balance between bias and variance, Focused intervals aim to achieve a good balance between the length of confidence intervals and the potential worst case asymptotic coverage over the likely space of direct instrument effects on the outcome.

### 4.1 Asymptotic distribution of the Focused estimator

A feature of the local mis-specification framework is that the sampling uncertainty from asymptotic bias estimation directly affects the asymptotic distribution of the post-selection Focused estimator $\hat{\theta }$. In particular, there is non-ignorable uncertainty in model selection: even if the use of only the core instruments is optimal from an AMSE perspective, the uncertainty in asymptotic bias estimation can cause the Focused estimator to erroneously select the full set of instruments.

In contrast, under consistent model selection, the Focused estimator would choose either ${\hat{\theta }}_{C}$ or ${\hat{\theta }}_{F}$ with probability approaching 1 as *n, p* → ∞. While this would seem to simplify the task of inference, it would still not be possible to consistently estimate the distribution of post-selection estimators uniformly over the space of direct effects *τ* (Leeb and Pötscher, 2005, Section 2.3, pp. 38–40). Moreover, consistent model selection does not suit our goal of improved estimation in terms of low risk, since the worst case risk of post-selection estimators would be unbounded (Leeb and Pötscher, 2008).

#### Theorem 3 (Focused estimator)

*Under Assumptions 1-4, the Focused estimator is consistent, and is asymptotically distributed* $\hat{\theta }\overset{a}{\sim }\theta_{0}+\Lambda \left(b\right)$ *as n, p* → ∞ *where* $\Lambda \left(b\right)=I\left\{W\left(b\right)\leq 0\right\}\left(b+K_{F}\right)+\left(1-I\left\{W\left(b\right)\leq 0\right\}\right)K_{C},W\left(b\right)=\max\left({\left(b+K_{b}\right)}^{2}-\Delta_{B},0\right)+\Delta_{F}-\Delta_{C}$, *and*

$$K≔\left[\begin{array}{c} K_{F}\\ K_{C}\\ K_{b} \end{array}\right]\sim N\left(\left[\begin{array}{c} 0\\ 0\\ 0 \end{array}\right],\Delta ≔\left[\begin{array}{c} \Delta_{F}\Delta_{E}\Delta_{D}\\ \Delta_{E}\Delta_{C}\Delta_{A}\\ \Delta_{D}\Delta_{A}\Delta_{B} \end{array}\right]\right),$$

*where* $\Delta_{E}=\eta_{C}^{-1}{\left(\eta_{C}+\eta_{S}\right)}^{-1}\left(\eta_{C}+\varsigma_{C}\right),\Delta_{A}=-\Delta_{E}\eta_{C}^{-1}\eta_{S}$, *and* $\Delta_{D}=\eta_{C}^{-1}{\left(\eta_{C}+\eta_{S}\right)}^{-2}\eta_{C}\left(\eta_{S}+\varsigma_{S}\right)-\Delta_{E}{\left(\eta_{C}+\eta_{S}\right)}^{-1}\eta_{S}$.

Theorem 3 indicates that the asymptotic distribution of $\hat{\theta }$ is a weighted average of the asymptotic distributions of ${\hat{\theta }}_{C}$ and ${\hat{\theta }}_{F}$, where the weights are random even as *n, p* → ∞. As a result, “naive” confidence intervals for $\hat{\theta }$ that ignore sampling uncertainty in instrument selection should not be reported. Such intervals can perform extremely poorly in practice, with coverage arbitrarily far below their nominal level.

Under the condition $p/n{\left\|\beta_{X}\right\|}_{2}^{2}=O\left(1\right)$ as *n, p* → ∞ from Assumption 3, the variance components Δ can be consistently estimated, and therefore to simplify notation we henceforth assume that Δ is known.

### 4.2 Focused confidence intervals with coverage constraints

There are two related problems that a valid inference procedure must solve. First, confidence intervals need to be widened to account for the model selection uncertainty introduced by focused instrument selection. Second, confidence intervals need to be re-centered if the focused estimator is asymptotically biased.

If the true asymptotic bias component *b* was known, inference would be straightforward: Theorem 3 could be used directly to simulate the distribution of Λ(*b*). However, *b* cannot be consistently estimated in our locally invalid instruments framework. For this setting, DiTraglia (2016) proposes two feasible inference procedures that consider the distribution of Λ(*b*) at values of *b* that are *plausible* given the observed data.

The “1-step” interval is based on the distribution of Λ(*b*) evaluated at $b=\hat{b}$, and it effectively assumes that the true value of *b* is equal to its asymptotically unbiased estimator $\hat{b}$. This is intuitive since $\hat{b}$ is in some sense the *most* plausible value of *b* given the data. It follows that if $\hat{b}$ is close to the true value of *b*, then the 1-step interval will have coverage that is close to its nominal level. Moreover, when the direct effects *τ* are small, simulation evidence from DiTraglia (2016) suggests that the 1-step interval has competitive coverage and can be shorter in length than the “Core” interval, which is the standard confidence interval of ${\hat{\theta }}_{C}$ that uses only the core instruments. More generally, however, the 1-step interval comes with no theoretical guarantees; it may substantially under-cover, although its performance is much better than that of a naive interval that ignores instrument selection uncertainty.

The reason why the 1-step interval may under-cover is that it fails to account for the uncertainty in the estimate $\hat{b}$ of *b*, thus potentially leading to intervals that are too short and centered incorrectly. The “2-step” interval allows for such uncertainty by first constructing a confidence region *φ* for *b*. It then simulates the distribution of Λ(*b*′) at every value *b*′ in *φ*, constructing a collection of confidence intervals each based on the assumption that the true value of *b* is *b*′. To obtain a uniform coverage guarantee, the 2-step interval takes the outer envelope of all of the resulting intervals. This makes the 2-step interval extremely conservative: in general there is *no* value of *b* for which the actual coverage equals the nominal coverage, and hence it will always over-cover. Our simulation evidence suggests that this over-coverage problem makes the 2-step intervals too wide to be useful in practice.

We consider a way forward for improved inference with “Focused” intervals. These intervals aim to combine the strengths of the 1-step and 2-step intervals while avoiding their drawbacks. Like the 1-step interval, it is constructed by simulating Λ(*b*) at a *single* value of *b* rather than taking an outer envelope over many values. This means that it can yield shorter confidence intervals. Like the 2-step interval, however, it comes with theoretical guarantees. The key is to choose an appropriate value of *b*.

The Focused interval considers only values of *b* that are contained in a (1 – *α*_1_) × 100% confidence interval called *φ*. This is the same confidence interval for *b* used in the 2-step interval approach. For some values of *b* in *φ* the distribution of Λ(*b*) will be highly dispersed. Suppose that *b*′ is such a value, so that a (1 – *α*_2_) × 100% confidence interval *CI*(*b*′) for *θ*_0_ computed under the assumption that *b* = *b*′ will be relatively wide. By construction, *CI*(*b*′) will achieve nominal coverage probability 1 – *α*_2_ when *b* = *b*′. The key insight is as follows: if *b*″ is a value in *φ* for which Λ(*b*″) is relatively less dispersed, then *CI*(*b*′) may also be a *nearly* valid confidence interval for *θ*_0_ when *b* = *b*″. Using this idea, the construction of the Focused interval proceeds as follows, based on a user-specified tolerance *γ* and nominal coverage probability 1 – *α*.

Algorithm 1 **(Focused interval with a minimum coverage constraint)**

***1.*** *Construct a* (1 – *α*_1_) × 100% *confidence interval φ for b using Theorem 2.*

***2.*** *For each b′* ∈ *φ, calculate a collection of* (1 – *α*_2_) × 100% *intervals* [*a_l_*(*b*′), *a_u_*(*b′*)] *for* Λ(*b′*) *using Theorem 3, each under the assumption that b* = *b*′.

***3.*** *Calculate* $\bar{\varphi }=\left\{b^{\prime }\left(\alpha ,\gamma \right)\in \varphi :P\left(a_{l}\left(b^{\prime }\right)\leq \Lambda \left(b^{\prime \prime }\right)\leq a_{u}\left(b^{\prime }\right)\right)\geq 1-\alpha_{2}-\gamma ,\text{for}\;\text{all}\;b^{\prime \prime }\in \varphi \right\}$.

***4.*** *Find the value of* $b^{\prime }\left(\alpha ,\gamma \right)\in \bar{\varphi }$ *that yields the shortest* (1 – *α*_2_) × 100% *confidence interval for* Λ(*b′*). Call this value b*(*α, γ*). *A CI for θ*_0_ *is then* $\left[\hat{\theta }-a_{l}\left(b*\left(\alpha ,\gamma \right)\right),\hat{\theta }-a_{u}\left(b*\left(\alpha ,\gamma \right)\right)\right]$.

***5.*** Notice that b* (*α, γ*) *depends on γ, α*_1_, *and α*_2_. *Repeat steps 1-4 for a range of choices of α*_1_ *subject to the constraint α*_1_ + *α*_2_ = *α. Choose the value of α*_1_ *that yields the shortest interval for θ*_0_.

Like the 1-step interval, the Focused interval is *always* shorter than the 2-step interval because it is one of the intervals contained in the outer envelope that forms the 2-step interval. Unlike the 1-step interval, its asymptotic coverage probability can never fall below 1 – *α* – *γ*.

**Fig 2:**
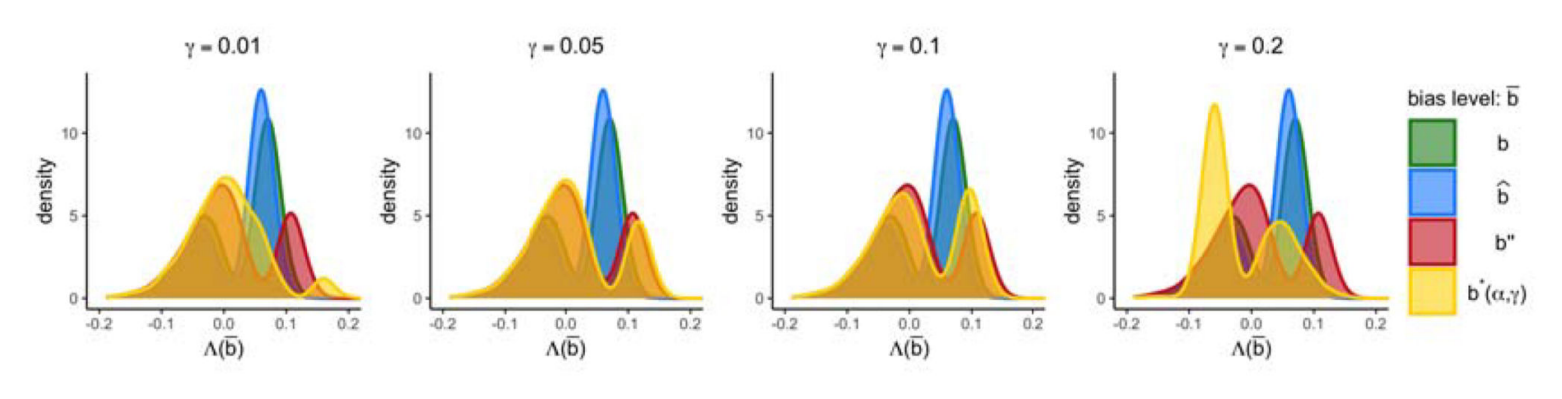
The distribution of $\Lambda \left(\bar{b}\right)$ evaluated at $\bar{b}=b,\bar{b}=\hat{b}$, and $\bar{b}=b*\left(\alpha ,\gamma \right)$, for the case where all instruments are equally strong, and the additional instruments are invalid.

To illustrate the differences between the 1-step, 2-step, and Focused intervals, Figure 2 plots the distributions of $\Lambda \left(\bar{b}\right)$ evaluated at different bias levels $\bar{b}$. The true bias is *b*, an asymptotically unbiased estimator for the bias is $\hat{b}$, while b*(*α, γ*) and *b″* are other bias levels contained in a (1 – *α*_1_) × 100% confidence region *φ* for *b*. Since *b* is unknown, we cannot directly calculate a confidence interval based on the distribution of Λ(*b*) given in green. The 1-step (1 – *α*) × 100% confidence interval is based on the distribution of $\Lambda \left(\hat{b}\right)$ given in blue. The 2-step interval takes an outer envelope of (1 – *α*_2_) × 100% confidence intervals based on the distributions of $\Lambda \left(\hat{b}\right)$ in blue, Λ(b*(*α, γ*)) in yellow, Λ(*b*″) in red, and all other distributions of $\Lambda \left(\bar{b}\right)$ for every $\bar{b}$ in *φ*.

The Focused interval is a (1 – *α*_2_) × 100% confidence interval based on the yellow distribution of Λ(b*(*α*, *γ*)) in Figure 2, and this interval also covers (1 – *α*_2_ – *γ*) × 100% of the distributions of $\Lambda \left(\hat{b}\right)$ and Λ(*b*″), and all other distributions $\Lambda \left(\bar{b}\right)$ for every $\bar{b}$ in *φ*. Note that the distribution of Λ(b*(*α, γ*)) that the Focused interval is based on is changing with the choice of *γ*. For lower values of *γ*, Λ(b*(*α, γ*)) is relatively dispersed: a bias level b*(*α*, *γ*) that corresponds to a more dispersed distribution of Λ(b*(*α, γ*)) is selected in order to satisfy the more stringent (1 – *α*_2_ – *γ*) × 100% coverage requirement of the distributions of $\Lambda \left(\bar{b}\right)$, for all $\bar{b}\in \varphi$.

#### Theorem 4 (Worst case asymptotic coverage of Focused intervals)

*Under Assumptions 1-4, the Focused interval defined in Algorithm 1 has asymptotic coverage probability no less than* 1 – *α* – *γ as n, p* → ∞.

The Focused interval is designed to achieve nominal coverage 1 ‒ *α* at a plausible value of the asymptotic bias, while also controlling the *worst case* asymptotic coverage according to a maximum allowable size distortion *γ*. The choice of *γ* dictates the trade off between the worst case coverage level and the length of the interval, with a lower level of tolerance *γ* more likely to provide conservative inference. The feasibility of constructing the Focused interval relies on the existence of a sufficiently dispersed distribution of Λ(*b′*(*α,γ*)) at a plausible value *b′*(*α,γ*), which may not exist for extremely low levels of *γ*. However, selecting an extremely low level of *γ* would defeat the purpose of the Focused interval, which aims to be competitive in terms of *both* coverage and length.

Although Algorithm 1 is novel, the concept of an inference procedure that depends on a user-specified allowable size distortion has precursors in the econometrics literature. For example, Andrews (2018) proposes an inference strategy that controls a worst case coverage distortion under weak instruments, and where the decision to report a conventional or weak instrument robust confidence set depends on the level of under-coverage that an investigator is willing to accept.

## 5 Simulation study

In this section we illustrate how the finite sample performance of the Focused estimator and Focused confidence interval depends on the strength of instruments and the magnitude of direct variant effects on the outcome.

First, we consider estimation performance in terms of root-mean squared error (RMSE). Second, we show how the Focused interval may be able to achieve a favourable balance of length and coverage, and discuss where it may lead to improved inference compared with the Core interval, which is the conventional confidence interval of ${\hat{\theta }}_{C}$ based on using only the core instruments. Third, we discuss the sensitivity of the Focused interval to the choice of *γ* which controls the worst case coverage loss. Finally, we consider the performance of the Focused estimator when the core instruments *S*_0_ are in fact invalid.

### 5.1 Design

We simulated two-sample summary data on *p* = 110 variants according to Assumptions 1–4. The sample sizes were set at *n* = *n_X_* = *n_Y_* = 1000. Of the 110 variants, 10 were set to be valid instruments, and they formed the core set *S*_0_. The remaining *p* – |*S*_0_| = 100 variants formed the set of additional instruments *S*.

We generated estimated associations ${\hat{\beta }}_{X_{j}}\sim N\left(\beta_{X_{j}},\sigma_{X_{j}}^{2}\right)$ and ${\hat{\beta }}_{Y_{j}}\sim N\left(\beta_{Y_{j}},\sigma_{Y_{j}}^{2}\right)$, where true genetic variant associations with the risk factor were set as $\beta_{X_{j}}={\bar{\beta }}_{C}/\sqrt{\left|S_{0}\right|}$ for *j* ∈ *S*_0_, and $\beta_{X_{j}}={\bar{\beta }}_{S}/\sqrt{p-\left|S_{0}\right|}$ for *j* ∈ *S*, where ${\bar{\beta }}_{C}$ and ${\bar{\beta }}_{S}$ were chosen to maintain a particular level of the *concentration parameters* $\lambda_{C}=\sum_{j\in S_{0}}\beta_{X_{j}}^{2}/\left(\left|S_{0}\right|\sigma_{X_{j}}^{2}\right)$ and $\lambda_{S}=\sum_{j\in S}\beta_{X_{j}}^{2}/\left(\left|S\right|\sigma_{X_{j}}^{2}\right)$, which are measures of the average instrument strength of *S*_0_ and *S*. The variances $\sigma_{X_{j}}^{2}$ and $\sigma_{Y_{j}}^{2}$ were set equal to 1/*n* for all variants.

The true variant–outcome associations were set to be *β_Y_j__* = *β_X_j__ θ*_0_ for *j* ∈ *S*_0_, and *β_Y_j__* = *β_X_j__ θ*_0_ + *τ_j_* for *j* ∈ *S*, where the true causal effect was *θ*_0_ = 0.2, and the direct effects are fixed effects generated as $\tau_{j}\sim U[0,\bar{\tau }/\sqrt{np}]$, for different values $\bar{\tau }\geq 0$.

For inference, we set the nominal coverage probability at 1 – *α* = 0.95, and unless otherwise stated, the allowable worst case size distortion for the Focused interval was set at *γ* = 0.2. Along with the Focused, 1-step, 2-step, and Core intervals described above, we also note the performance of the “Naive” confidence interval, which is the standard confidence interval for the selected estimator that ignores sampling uncertainty in model selection.

### 5.2 Estimation

**Fig 3:**
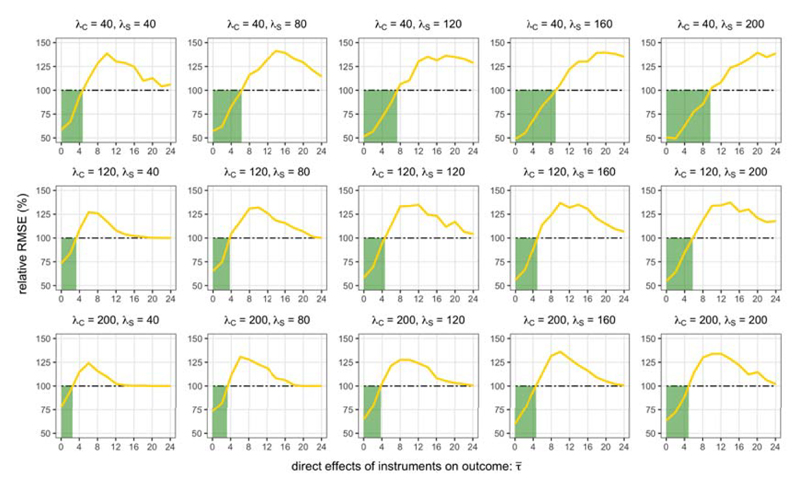
RMSE of Focused estimator relative to RMSE of Core estimator varying with the average instrument strength of S_0_ (λ_C_) and S (λ_S_), and invalidness of $S\left(\bar{\tau }\right)$.

Figure 3 highlights that when the direct variant effects on the outcome are sufficiently small, the RMSE of the Focused estimator is lower than the Core estimator. However, this improvement is not uniform across larger values of $\bar{\tau }$. The performance of the Focused estimator worsens for more intermediate values of $\bar{\tau }$. Then, as $\bar{\tau }$ becomes large, the Focused estimator should select the Core estimator with large probability, so that the RMSEs of the Core and Focused estimators should be quite similar.

The extent of the improvement in RMSE that is possible appears to depend on the strength of instruments. For example, when $\bar{\tau }=2$, the Focused estimator offered a 32.8% reduction in RMSE when all instruments are relatively weak (λ*_C_* = λ*_S_* = 40), a 30.7% reduction when λ*_C_* = λ*_S_* = 120, and a 27.8% reduction when all instruments are relatively strong (λ*_C_* = λ*_S_* = 200).

The relative strengths of the core and additional instrument sets affect the values of $\bar{\tau }$ over which focused instrument selection is able to improve estimation. When the additional instruments were strong and the core instruments were relatively weak (λ*_C_* = 40, λ*_S_* = 200), the Focused estimator had a lower RMSE than the Core estimator over the range $0\leq \bar{\tau }<10$. In contrast, when λ*_C_* = 200 and λ*_S_* = 40, the Focused estimator had a lower RMSE only over the range $0\leq \bar{\tau }\leq 2$.

In summary, these estimation results intuitively suggest that focused instrument selection is more likely to improve estimation when the additional instruments are not too invalid, the core instruments are quite weak, and the additional instruments are strong. This is practically relevant for MR analyses of polygenic traits where several genes may be causally related.

### 5.3 Confidence intervals

**Fig 4:**
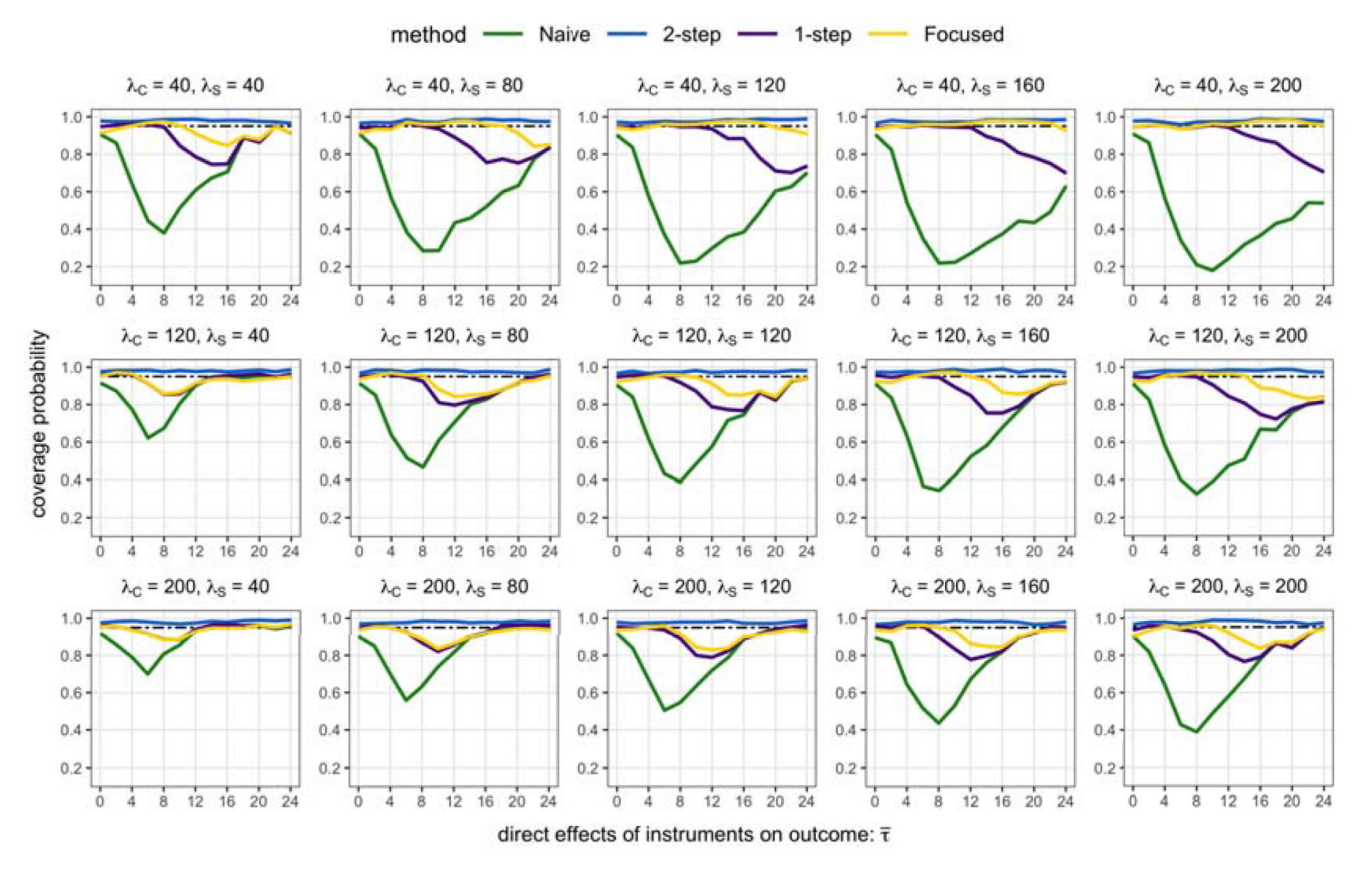
Coverage probabilities of confidence intervals (nominal coverage is 1 – *α* = 0.95).

**Fig 5:**
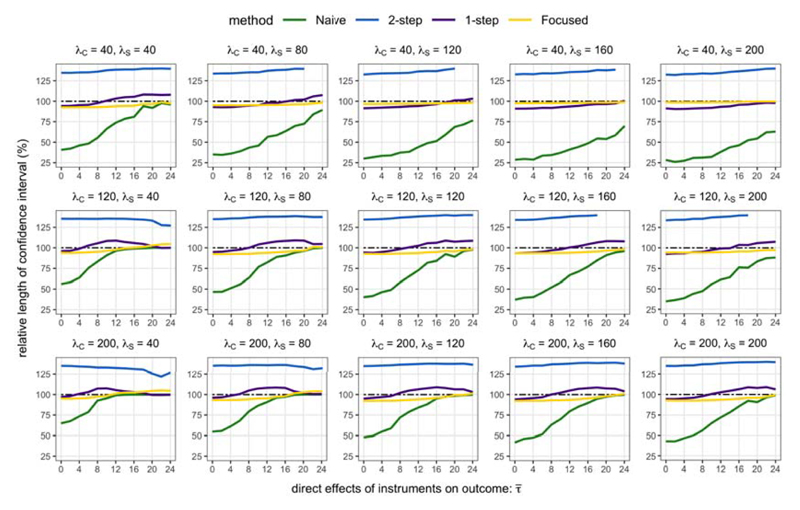
Length of confidence intervals relative to the Core interval (nominal coverage is 1 – *α* = 0.95).

For inference, Figure 4 shows that the coverage probability of the Naive interval dropped to as low as 0.4 when all instruments are equally strong $\left(\bar{\tau }=8\right)$, and lower than 0.2 when the additional instruments are much stronger than the core instruments (λ*_C_* = 40, λ*_S_* = 200, $\bar{\tau }=10$). This underscores the importance for confidence intervals of $\hat{\theta }$ to account for sampling uncertainty in model selection. On the other hand, the 2-step intervals are conservative; the intervals exceeded nominal coverage probability, and Figure 5 shows that they were generally over 30% longer in length than the Core intervals.

The 1-step intervals are a useful compromise between the 2-step and Naive intervals. When the additional instruments were not too invalid (i.e. for small enough parameter values of $\bar{\tau }$), the 1-step intervals were shorter in length than the Core intervals, while also achieving nominal coverage probability. The performance of the 1-step interval appears to be quite sensitive to the relative strengths of the core and additional instruments. When the additional instruments were relatively strong (for example, λ*_C_* = 40, λ*_S_* ≥ 160), the 1-step intervals were shorter than the Core intervals, but they under-covered for large enough values of $\bar{\tau }$. Conversely, when the core instruments are strong enough, the 1-step intervals showed no real advantages compared with the Core interval.

For the Focused intervals, we selected the allowable worst case size distortion as *γ* = 0.2, so that for a 1 – *α* = 0.95 level confidence interval, coverage probability should not be lower than 0.75. Figure 4 shows that the coverage of the Focused interval was higher than 0.8 for all parameter values $\bar{\tau }$, whereas for some values the coverage probability of the 1-step interval dropped below 0.7. Moreover, the coverage of the Focused interval was generally more competitive than the 1-step interval, especially when the additional instruments were at least as strong as the core instruments.

When the core instruments were not much weaker than the additional instruments, the Focused intervals were shorter in length than the 1-step interval. Interestingly, the Focused intervals were also up to 8% shorter in length than the Core intervals unless the additional instruments were very invalid $\left(\bar{\tau }\geq 12\right)$. The Focused interval can therefore improve the power of an analysis by incorporating information from additional relevant instruments if they are not too invalid. At the same time, the Focused interval also retains good size control and buys insurance against serious under-coverage when additional instruments are very invalid.

### 5.4 Sensitivity to the choice of γ

**Fig 6:**
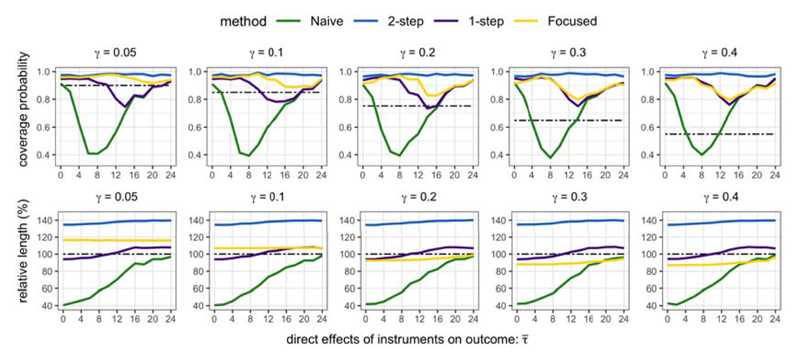
The dashed line in the first row is the allowable size distortion 1 – *α* – *γ* (nominal coverage is 1 – *α* = 0.95). The second row plots the length of confidence intervals relative to the Core interval.

The Focused intervals require investigators to choose an acceptable level of the worst case size distortion *γ*. Figure 6 illustrates how the performance of the Focused interval varies according to the choice of *γ* for the case where all instruments are equally strong (λ*_C_* = λ*_S_* = 40). The results of other confidence intervals discussed in this paper are also shown for comparison, but of course their performance should not vary with *γ*.

Figure 6 verifies that the Focused interval is able to control the worst case coverage over all parameter values $\bar{\tau }$. The cost of allowing only a small size distortion is the longer length of the interval. The Focused intervals were over 15% longer in length than the Core intervals when *γ* = 0.05, although they were also much shorter than the 2-step intervals.

Conversely, for larger values of *γ*, the Focused intervals become shorter, but only to a certain degree: since the Focused intervals account for uncertainty in model selection, the lengths of the intervals are not as short as the Naive intervals. Accordingly, the coverage probability of the Focused intervals will also not change for large enough values of *γ*.

### 5.5 Estimation when S_0_ contains invalid instruments

**Fig 7:**
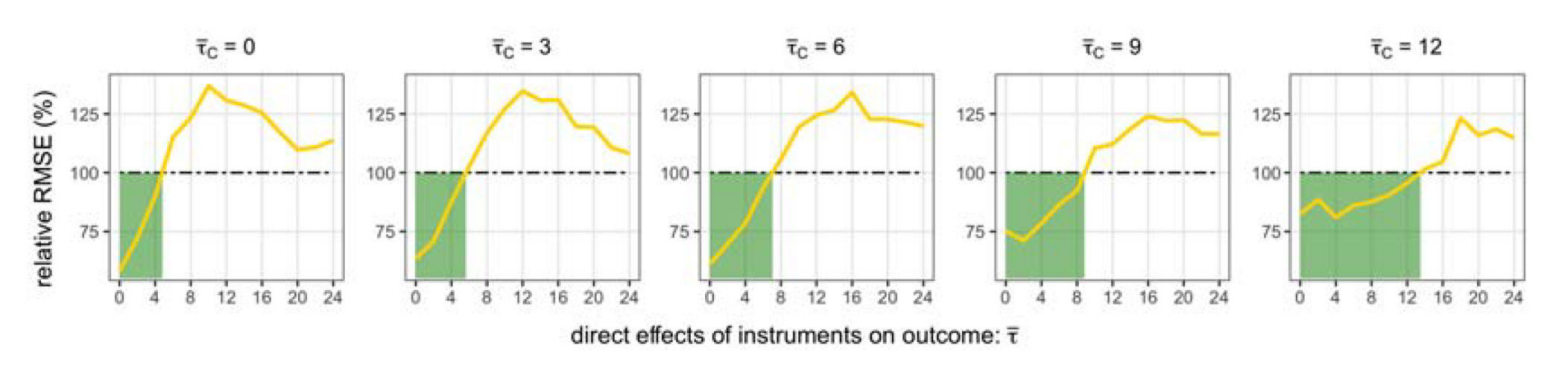
RMSE of Focused estimator relative to RMSE of Core estimator varying with the invalidness of $S_{0}\left({\bar{\tau }}_{C}\right)$ and invalidness of $S\left(\bar{\tau }\right)$.

Focused instrument selection aims to prioritise the evidence suggested by a core set of genetic variants *S*_0_ that are believed to be valid instruments. In practice, investigators may not always be correct in their belief, and *S*_0_ may actually consist of invalid instruments. We consider this setting in simulation by slightly altering the design in Section 5.1 so that the true variant–outcome associations are set to be *β*_Y_j__** = *β_X_j__θ*_0_ + *τ_j_*, where $\tau_{j}\sim U\left[0,{\bar{\tau }}_{C}/\sqrt{np}\right]$ for *j* ∈ *S*_0_. Figure 7 presents the estimation results for the case where all instruments are equally strong (λ*_C_* = λ*_S_* = 40).

Our results show that when *S*_0_ contained only slightly invalid instruments, the Focused estimator was able to improve on the Core estimator in terms of RMSE. As the instruments in *S*_0_ become more invalid, but not as invalid as *S*, the performance of the Focused estimator worsens because the estimated bias from including *S* is under-estimated. We note that the Focused estimator also performed relatively well when the instruments *S*_0_ were at least as invalid than *S*; for example, the RMSE of the Focused estimator was lower than that of the Core estimator when ${\bar{\tau }}_{C}=\bar{\tau }=12$.

## 6 Empirical Examples

In this section, we demonstrate how focused instrument selection can be applied in MR studies. First, we consider the problem of instrument selection in *drug target MR,* where variation in a gene that encodes the protein target of a drug is used to proxy drug target perturbation. Such MR investigations have the potential to provide genetic evidence on drug efficacy and to study potential side effects (Gill et al., 2021).

An important decision in drug target MR is to specify a “*cis* window” that defines a gene region from which instruments are selected. In practice, investigators often use *cis* windows that are wider than gene regions defined by tools such as GeneCards (Stelzer et al., 2016) in order to boost the power of an analysis through the use of multiple instruments. This leaves the possibility of a type of publication bias where researchers may report findings only from a *cis w*indow that gives their preferred result. In Section 6.1, we apply the focused instrument selection method to two lipid drug targets, where genetic variants that may be included only from widening a *cis* window are additional instruments.

Second, we investigate the effect of vitamin D supplementation on a range of outcomes. GWASs have identified strong genetic associations in biologically plausible genes known to have a functional role in the transport, synthesis, or metabolism of vitamin D. Some previous MR studies aiming to study vitamin D effects have used genetic variants located in neighborhoods of those genes to instrument vitamin D as they are considered more likely to satisfy the exclusion restriction than other genome-wide significant variants (Mokry et al., 2015; Revez et al., 2020). However, the role of many other genes which are robustly associated with vitamin D may not yet be fully understood; for example, Jiang, Ge and Chen (2021) selected genetic variants from 69 independent loci to instrument vitamin D. In Section 6.2, we use genetic variants from biologically plausible genes as the core set of instruments, and apply focused instrument selection to select from many additional genetic variants which may be considered more likely to have a direct effect on the outcome through their effects on traits other than vitamin D.

For Focused confidence intervals, we selected *γ* = 0.2 for the maximum allowable size distortion. The data used for our analyses are publicly available through the MR-Base platform (Hemani et al., 2018), and R code to perform our empirical investigation is available on GitHub at github.com/ash-res/focused-MR/.

### 6.1 CETP and PCSK9 inhibitors

Cholesteryl ester transfer protein (CETP) inhibitors are a class of drugs that raise high density lipoprotein cholesterol levels and lower low density lipoprotein cholesterol (LDL-C) levels. At least three CETP inhibitors have failed in clinical trials to conclude a protective effect against coronary heart disease (CHD), but the successful trial of Anacetrapib showed a modest benefit when used with statins (Bowman et al., 2017). A recent drug target MR analysis by Schmidt et al. (2021) offers genetic evidence that CETP inhibition may be an effective approach for preventing CHD. Here we investigate the robustness of a similar drug target MR study to the choice of a *cis* window used to select instruments.

We study the genetically predicted LDL-C lowering effect of CETP inhibition on a range of outcomes by using genetic variants located in a neighborhood of the *CETP* gene. We may consider instruments drawn from the “narrow” *cis* window Chr 16: bp: 56,985,862–57,027,757 (which is the region stated in GeneCards ±10,000 bp) to more accurately represent the genetically predicted effects of CETP inhibition. At the same time, it is also common for drug target MR studies to use a “wider” *cis* window for instrument selection. We may consider potential additional instruments from the wider window Chr 16: bp: 56,895,862–57,117,757 (which is the region stated in GeneCards ±100,000 bp). The use of these additional instruments may be considered more likely to lead to biased estimation compared with using only those variants from the narrow *cis* window.

Therefore the Core estimator used only the 3 uncorrelated genetic variants located in the narrow window as instruments, while the number of additional variants that were available varied between 4 and 10 depending on the outcome of interest. The Focused estimator selected the full set of instruments for 12 out of the 16 outcomes we studied. The concentration parameter for the core instruments was 91.10, while the concentration parameter for the additional instruments was between 6.52 and 8.12 depending on how many additional instruments were available for the application.

**Fig 8:**
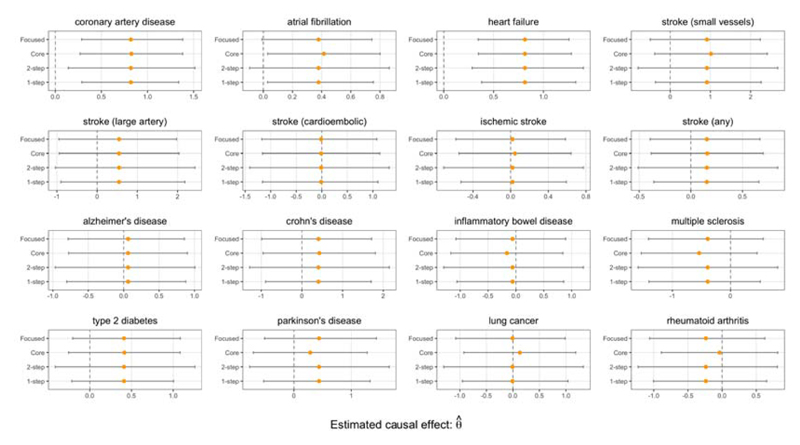
CETP gene analysis. Point estimates and 95% confidence intervals of the change in log odds ratio of various outcomes due to a 1 standard deviation increase in instrumented LDL-C.

From Figure 8, we find that the genetically predicted LDL-C lowering effect of CETP inhibition is associated with a lower risk of CHD. The results also suggest that genetically predicted CETP inhibition may have a protective effect on other cardiovascular disease outcomes; specifically atrial fibrillation and heart failure. We do not find evidence for a protective effect on stroke outcomes, nor do we find evidence for any adverse effects on various non-cardiovascular related outcomes.

Compared with CETP inhibitors, PCSK9 inhibitors (PCSK9i) are a more established class of drugs that lower LDL-C levels. A drug target MR analysis can genetically proxy the effect of taking PCSK9i by instrumenting LDL-C using genetic variants located in a neighborhood of the *PCSK9* gene. Similar to the *CETP* gene analysis above, we consider variants located in a narrow *cis* window of *PCSK9* (Chr 1: bp: 55,505,221–55,530,525; exactly equal to the window stated in GeneCards) as core instruments, while additional variants taken from a wider window ±100,000 bp are additional instruments.

Using this criteria, there were 4 core instruments when studying the same outcomes as before, apart from the case of lung cancer where there were 3. The number of additional instruments available ranged from 2 to 13. The Focused estimator again selected the full set of instruments for 12 out of 16 outcomes. The set of core instruments was very strong compared with the additional instruments; the concentration parameter for the core instruments was 618.71, compared with a range of 8.72 to 26.63 for the full set of additional variants available.

**Fig 9:**
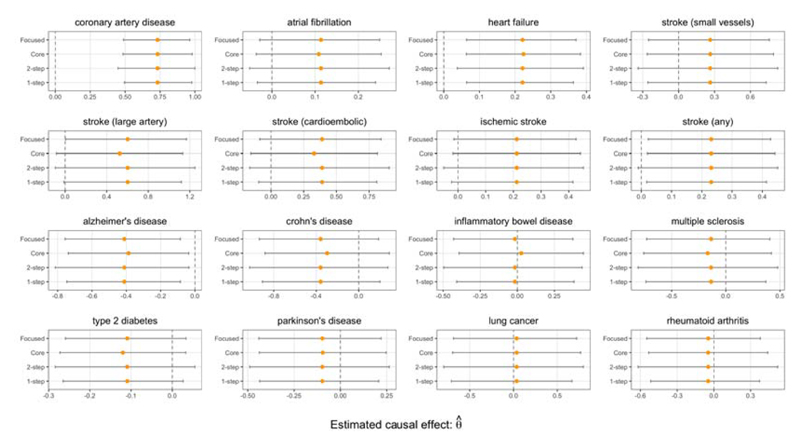
PCSK9 gene analysis. Point estimates and 95% confidence intervals of the change in log odds ratio of various outcomes due to a 1 standard deviation increase in instrumented LDL-C.

The results in Figure 9 suggest that the genetically predicted LDL-C lowering effect of PCSK9i is associated with a lower risk of coronary artery disease, heart failure, and stroke incidence; in addition, the Focused intervals suggest an association specifically with large artery stroke incidence. We also find genetic evidence that PCSK9 inhibition may adversely affect the risk of developing Alzheimer’s disease, which supports the findings of Williams et al. (2020) and Schmidt et al. (2021).

### 6.2 Vitamin D supplementation

Finally, we apply focused instrument selection to estimate the genetically predicted effect of vitamin D supplementation on a range of outcomes. Previous MR studies instrumenting vitamin D have used variants from genes implicated in the modulation of 25OHD levels through known mechanisms. In particular, the GC, *DHCR7, CYP2R1* and *CYP24A1* genes have known functions in vitamin D transport, synthesis, or metabolism (Berry et al., 2012; Mokry et al., 2015). Therefore we take genetic variants from neighborhoods of these genes (±500,000 bp on regions stated in GeneCards) as our set of core instruments.

Moreover, GWASs also provide data on many other robustly associated genetic variants with vitamin D (Jiang, Ge and Chen, 2021). We use other variants passing a genome-wide significance threshold (p-value association with vitamin D less than 5 × 10^−8^) to form additional instrument sets. Instead of choosing between the Core and Full estimator, in our analysis of vitamin D effects we allowed the Focused estimator to also choose from subsets of the additional instruments. We partitioned the additional instruments into 3 groups by k-means clustering based on the ratio estimate of each variant $\left({\hat{\beta }}_{Y_{j}}/{\hat{\beta }}_{X_{j}},j\in S\right)$ and then considered all possible combinations of these 3 partitions, thus creating 7 sets of additional instruments.

In nearly all the outcomes we considered, there were between 10 and 11 genome-wide significant variants from the GC, *DHCR7, CYP2R1* and *CYP24A1* gene regions which were used as core instruments for vitamin D. For the outcomes primary biliary cirrhosis and asthma there were only 4 variants available to use as core instruments. Many additional instruments (≥ 50) were selected by the Focused estimator in all cases. The core instruments were again stronger than the additional instruments; for the core instruments the concentration parameter ranged from 625.52 to 723.21, and for the additional instruments it ranged from 49.73 to 99.80.

**Fig 10:**
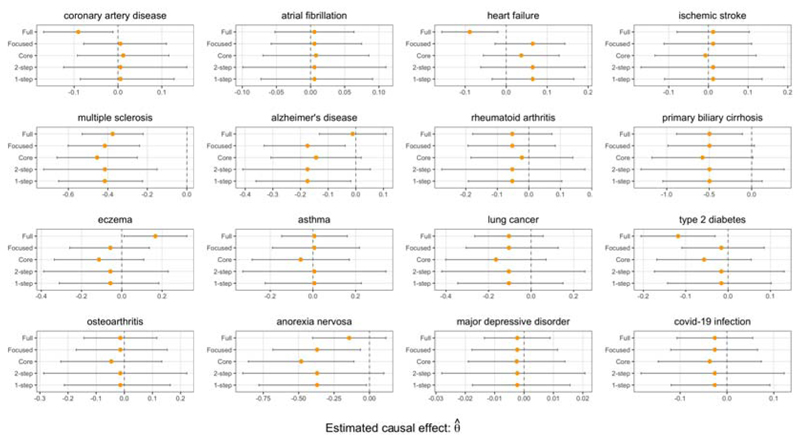
Vitamin D effects. Point estimates and 95% confidence intervals of the change in log odds ratio of various outcomes due to a 1 standard deviation increase in instrumented vitamin D levels.

Evidence from observational studies suggests that low serum vitamin D levels are associated with an increased risk of cardiovascular disease (Dobnig et al., 2008). These reported associations may be due to unmeasured confounding, as evidence from a meta-analysis of 21 randomized clinical trials suggests no causal link (Barbarawi et al., 2019). From Figure 10, we find no genetically predicted effect of vitamin D on cardiovascular outcomes.

Our analysis is able to highlight that using the full set of available instruments can sometimes lead to very different estimates than when the core instruments are prioritised. In particular, the standard confidence intervals of the Full estimator suggest a non-null association of vitamin D on coronary artery disease, heart failure, eczema, primary biliary cirrhosis, and type 2 diabetes, but these results are not supported by the Focused intervals.

Our results suggest that higher vitamin D level may have a protective effect on the incidence of multiple sclerosis, a finding which has previously been discussed in other MR studies (Mokry et al., 2015). Through the Core and Focused intervals, we also find that genetically predicted vitamin D level may be associated with anorexia.

The 1-step and Focused intervals of the Focused estimator suggest that higher vitamin D level may have a protective effect on the risk of developing Alzheimer’s disease, which supports the findings from Jiang, Ge and Chen (2021), but interestingly this is not supported by the Core and Full intervals. The Core estimator used 11 instruments, the Full estimator used 91 additional instruments, and the Focused estimator selected a subset of 65 of the additional instruments. This illustrates the ability of the focused instrument selection method to carefully select additional instruments that can potentially improve the power of an analysis.

## 7 Conclusion

Publicly available GWAS summary data have revealed that hundreds of genetic variants are robustly associated with a wide range of traits and diseases. However, MR studies in practice do not always make use of all genetic variants that are strongly associated with risk factors of interest; in some applications, investigators may have greater confidence in the instrument validity of only a smaller subset of many genetic variants. For this setting, we propose a way forward for improved estimation through *focused* use of many weak and potentially invalid instruments.

Whether focused use of invalid instruments can in turn improve inference is an open question. While a uniform improvement is not possible, we propose a new strategy for postselection inference through Focused intervals, which are shown to achieve a good balance between precision and coverage probability while also guarding against a user-specified worst case size distortion. Our empirical applications highlight the potential of focused instrument selection to uncover new causal relationships in MR studies.

For neither estimation nor inference do we obtain uniform improvements, but such results could be explored under different settings. For multivariable Mendelian randomization where we are interested in estimating the effects *θ*_0_ = (*θ*_1_,…, *θ_K_*)′ that multiple exposures may have on an outcome, it would be interesting to consider uniformly improved estimation in terms of an aggregate risk function, in similar spirit to Rosenman et al. (2023). Compared with the usual frequentist notion of inference, uniform improvement in terms of both length and coverage is possible from the perspective of empirical Bayes (Casella and Hwang, 2012, p.56) or “average coverage” (Armstrong, Kolesár and Plagborg-Møller, 2022) criterions. Future work could explore empirical Bayes confidence intervals under quite general restrictions on the direct effects *τ_j_*, *j* ∉ *S*_0_.

## Supplementary Material

**Supplementary Material A**

This contains proofs of theoretical results, and further simulation results.

**Supplementary Material B**

This contains R code to apply the focused instrument selection method in two-sample Mendelian randomization studies.

Supplementary material
