# Supplementary material for: Selecting Invalid Instruments to Improve Mendelian Randomization with Two-Sample Summary Data
Source: Ann Appl Stat. Author manuscript; Available in PMC 2024 Jun 1. (PMC7615940; doi:10.1214/23-AOAS1856)
Supplement: Supplementary material [file EMS195813-supplement-Supplementary_material.pdf]

SUPPLEMENT TO "SELECTING INVALID INSTRUMENTS TO IMPROVE MENDELIAN RANDOMIZATION WITH TWO-SAMPLE SUMMARY DATA"

We prove general versions of Theorems 1–4 that allow a more flexible instrument selection choice. In particular, instead of simply choosing between the core set of instruments S_0 and the full set of instruments, we allow a choice between several additional instrument sets $S_1, \ldots, S_K$. The results stated in the main text can be obtained as corollaries for the case $K = 1$. Let S denote any generic set of additional instruments from $S_1, \ldots, S_K$, and let $\hat{\theta}_S$ denote the LIML estimator that uses the set of genetic variants $S_0 \cup S$ as instruments.

Let CS denote 'the Cauchy-Schwarz inequality', CH denote 'Chebyshev's inequality', and T denote 'the triangle inequality'. For all variants j, let $e_{Y_j} = \hat{\beta}_{Y_j} - \beta_{Y_j}$, $e_{X_j} = \hat{\beta}_{X_j} - \beta_{X_j}$, $\hat{g}_j(\theta) = \hat{\beta}_{Y_j} - \theta\hat{\beta}_{X_j}$, $\Omega_j(\theta) = \sigma^2_{Y_j} + \theta^2\sigma^2_{X_j}$, and $\Omega_j = \Omega_j(\theta_0)$.

Lemma S.1. (Consistency of $\hat{\theta}_S$). Under Assumptions 1-4, for any additional instrument set S, $\hat{\theta}_S - \theta_0 \xrightarrow{P} 0$ as $n, p \to \infty$.

Proof. Some simple algebra shows that $\hat{g}_j(\theta) = (e_{Y_j} - \theta e_{X_j}) + \beta_{X_j}(\theta_0 - \theta) + \tau_j$, so that for $\hat{Q}(\theta) = -2^{-1}\sum_{j\in S_0\cup S}\Omega_j(\theta)^{-1}\hat{g}_j(\theta)^2$,

$$\begin{aligned}
-2\hat{Q}(\theta) \;=\; & (\theta_0-\theta)^2\sum_{j\in S_0\cup S}\Omega_j(\theta)^{-1}\beta^2_{X_j} + p + \sum_{j\in S_0\cup S}\Omega_j(\theta)^{-1}[(e_{Y_j}-\theta e_{X_j})^2 - \Omega_j(\theta)] \\
& + \sum_{j\in S_0\cup S}\Omega_j(\theta)^{-1}\tau_j^2 + 2(\theta_0-\theta)\sum_{j\in S_0\cup S}\Omega_j(\theta)^{-1}\beta_{X_j}(e_{Y_j}-\theta e_{X_j}) \\
& + 2\sum_{j\in S_0\cup S}\Omega_j(\theta)^{-1}\tau_j(e_{Y_j}-\theta e_{X_j}) + 2(\theta_0-\theta)\sum_{j\in S_0\cup S}\Omega_j(\theta)^{-1}\beta_{X_j}\tau_j \\
:= \; & (\theta_0-\theta)^2\sum_{j\in S_0\cup S}\Omega_j(\theta)^{-1}\beta^2_{X_j} + p + R_{1p} + R_{2p} + R_{3p} + R_{4p} + R_{5p}.
\end{aligned}$$

Note that $E[R_{1p}] = 0$ and $Var(R_{1p}) = 2p$. Hence, $R_1 = O_P(\sqrt{p})$ by CH. Similarly, $R_{3p} = O_P(\sqrt{n}\|\beta_X\|_2 \cdot |\theta_0 - \theta|)$, and $R_{4p} = O_P(1)$. By CS, $R_{2p} = O(1)$ and $R_{5p} = O_P(\sqrt{n}\|\beta_X\|_2 \cdot |\theta_0 - \theta|)$. Thus,

$$-2\hat{Q}(\theta) = (\theta_0-\theta)^2\sum_{j\in S_0\cup S}\frac{\beta^2_{X_j}}{\sigma^2_{Y_j}+\theta^2\sigma^2_{X_j}} + p + O_P(\sqrt{p} + \sqrt{n}\|\beta_X\|_2\cdot|\theta_0-\theta|).$$

The rest of the proof is then identical to the Proof of Theorem 3.1 of Zhao et al. (2020, p.41). □

For Lemma S.2-S.4, let $\psi_j(\theta) = \Omega_j(\theta)^{-2}(\hat{\beta}_{Y_j} - \theta\hat{\beta}_{X_j})(\hat{\beta}_{X_j}\sigma^2_{Y_j} + \theta\hat{\beta}_{Y_j}\sigma^2_{X_j})$, for each variant j.

Lemma S.2. Under Assumptions 1-4, $\sum_{j\in S_0\cup S}\nabla_\theta\psi_j(\theta_0)/(\eta_C + \eta_S) \xrightarrow{P} -1$ as $n, p \to \infty$.

Proof. The first-order condition is given by $\sum_{j\in S_0\cup S}\psi_j(\hat{\theta}_S) = 0$. Also,

$$\begin{aligned}
\nabla_\theta\psi_j(\theta) = & -\hat{\beta}_{X_j}(\hat{\beta}_{X_j}\sigma^2_{Y_j} + \theta\hat{\beta}_{Y_j}\sigma^2_{X_j})\Omega_j(\theta)^{-2} + \hat{\beta}_{Y_j}\sigma^2_{X_j}(\hat{\beta}_{Y_j} - \theta\hat{\beta}_{X_j})\Omega_j(\theta)^{-2} \\
& -4\theta\sigma^2_{X_j}(\hat{\beta}_{Y_j} - \theta\hat{\beta}_{X_j})(\hat{\beta}_{X_j}\sigma^2_{Y_j} + \theta\hat{\beta}_{Y_j}\sigma^2_{X_j})\Omega_j(\theta)^{-3}
\end{aligned}$$

where

$$E[\nabla_\theta \psi_j(\theta_0)] = -\Omega_j^{-1}\beta_{X_j}^2 + \Omega_j^{-2}\sigma_{X_j}^2\tau_j^2 - 4\theta_0\Omega_j^{-2}\sigma_{X_j}^2\beta_{X_j}\tau_j - 4\theta_0^2\Omega_j^{-3}\sigma_{X_j}^4\tau_j^2.$$

Let $\phi_j(\theta) = \nabla_\theta\psi_j(\theta) - E[\nabla_\theta\psi_j(\theta)]$. Then,

$$\begin{aligned}
\phi_j(\theta_0) = &-\Omega_j^{-1}\beta_{X_j}e_{X_j} - \Omega_j^{-2}\sigma_{Y_j}^2\beta_{X_j}e_{X_j} - \theta_0\Omega_j^{-2}\sigma_{X_j}^2\beta_{X_j}e_{Y_j} - \Omega_j^{-2}\sigma_{Y_j}^2(e_{X_j}^2 - \sigma_{X_j}^2)\\
&-\theta_0\Omega_j^{-2}\sigma_{X_j}^2\tau_j e_{X_j} - \theta_0\Omega_j^{-2}\sigma_{X_j}^2 e_{Y_j}e_{X_j} + \theta_0\Omega_j^{-2}\sigma_{X_j}^2\beta_{X_j}e_{Y_j} - \theta_0^2\Omega_j^{-2}\sigma_{X_j}^2\beta_{X_j}e_{X_j}\\
&+\Omega_j^{-2}\sigma_{X_j}\tau_j e_{Y_j} - \theta_0\Omega_j^{-2}\sigma_{X_j}^2\tau_j e_{X_j} + \Omega_j^{-2}\sigma_{X_j}^2(e_{Y_j}^2 - \sigma_{Y_j}^2) - \theta_0\Omega_j^{-2}\sigma_{X_j}^2 e_{Y_j}e_{X_j}\\
&+\Omega_j^{-2}\sigma_{X_j}^2\tau_j e_{Y_j} - 4\theta_0\Omega_j^{-2}\sigma_{X_j}^2\beta_{X_j}e_{Y_j} - 4\theta_0\Omega_j^{-3}\sigma_{X_j}^2\sigma_{Y_j}^2 e_{Y_j}e_{X_j} - 4\theta_0^2\Omega_j^{-3}\sigma_{X_j}^4\tau_j e_{Y_j}\\
&-4\theta_0^2\Omega_j^{-3}\sigma_{X_j}^4(e_{Y_j}^2 - \sigma_{Y_j}^2) + 4\theta_0^2\Omega_j^{-2}\sigma_{X_j}^2\beta_{X_j}e_{X_j} + 4\theta_0^2\Omega_j^{-3}\sigma_{Y_j}^2\sigma_{X_j}^2(e_{X_j}^2 - \sigma_{X_j}^2)\\
&+4\theta_0^3\Omega_j^{-3}\sigma_{X_j}^4\tau_j e_{X_j} + 4\theta_0^3\Omega_j^{-3}\sigma_{X_j}^4 e_{Y_j}e_{X_j} - 4\theta_0\Omega_j^{-3}\sigma_{Y_j}^2\sigma_{X_j}^2\tau_j e_{X_j} - 4\theta_0^2\Omega_j^{-3}\sigma_{X_j}^4\tau_j e_{Y_j}\\
&\equiv \sum_{l=1}^{23}\phi_j^{(l)}(\theta_0).
\end{aligned}$$

First, $Var(\phi_j^{(1)}(\theta_0)) = \Omega_j^{-2}\beta_{X_j}^2\sigma_{X_j}^2$, so that $\sum_{j\in S_0\cup S} Var(\phi_j^{(1)}(\theta_0)) = \Theta(n\|\beta_X\|_2^2)$. Similarly, for each $l \in [23]$, we have that $\sum_{j\in S_0\cup S} Var(\phi_j^{(l)}(\theta_0)) = O(n\|\beta_X\|_2^2) + O(p)$. Therefore, by CH,

$$\begin{aligned}
P\Big(\Big|\frac{1}{\eta_C+\eta_S}\sum_{j\in S_0\cup S}\phi_j(\theta_0)\Big| > \kappa\Big) &\le \frac{1}{\kappa^2(\eta_C+\eta_S)^2}\Big(\sum_{j\in S_0\cup S} Var(\phi_j(\theta_0)) + 2\sum_{j\in S_0\cup S}\sum_{k\neq j} Cov(\phi_j(\theta_0),\phi_k(\theta_0))\Big)\\
&\le O(1/n\|\beta_X\|_2^2) + O(p/n^2\|\beta_X\|_2^4)\\
&= o(1),
\end{aligned}$$

where the second inequality uses $Cov(\phi_j(\theta_0),\phi_k(\theta_0)) \le Var(\phi_j(\theta_0))^{\frac{1}{2}}Var(\phi_k(\theta_0))^{\frac{1}{2}}$ and $\eta_C + \eta_S = \Theta(n\|\beta_X\|_2^2)$, and the equality follows by Assumption 3. Therefore,

$$\frac{1}{\eta_C+\eta_S}\sum_{j\in S_0\cup S}\nabla_\theta\psi_j(\theta_0) = \frac{1}{\eta_C+\eta_S}\sum_{j\in S_0\cup S}E[\nabla_\theta\psi_j(\theta_0)] + o_P(1).$$

and

$$\begin{aligned}
\frac{1}{\eta_C+\eta_S}\sum_{j\in S_0\cup S}E[\nabla_\theta\psi_j(\theta_0)] &= -1 + \frac{1}{\eta_C+\eta_S}\Big[\sum_{j\in S_0\cup S}\Omega_j^{-2}\sigma_{X_j}^2\tau_j^2 - 4\theta_0\sum_{j\in S_0\cup S}\Omega_j^{-2}\sigma_{X_j}^2\beta_{X_j}\tau_j\\
&\quad -4\theta_0^2\sum_{j\in S_0\cup S}\Omega_j^{-3}\sigma_{X_j}^4\tau_j^2\Big]\\
&= -1 + \frac{1}{\eta_C+\eta_S}[O(n\|\tau\|_2^2) + O(n\|\beta_X\|_2\|\tau\|_2)]\\
&= -1 + O(1/n\|\beta_X\|_2^2) + O(1/\sqrt{n}\|\beta_X\|_2)\\
&= -1 + o_P(1).
\end{aligned}$$

Hence, by T, $\sum_{j\in S_0\cup S}\nabla_\theta\psi_j(\theta_0)/(\eta_C+\eta_S) = -1 + o_P(1)$. □

Lemma S.3. Under Assumptions 1-4,

$$\sum_{j\in S_0\cup S}\psi_j(\theta_0)/\sqrt{\eta_C+\eta_S+\varsigma_C+\varsigma_S} \xrightarrow{D} N(b_S/\sqrt{\eta_C+\eta_S+\varsigma_C+\varsigma_S}, 1)$$

as $n, p \to \infty$.

Proof. We can decompose $\psi_j(\theta_0)$ into a fixed bias term b_j, and stochastic terms J_{1j} and J_{2j},

$$\psi_j(\theta_0) = b_j + J_{1j} + J_{2j},$$

where $b_j = \Omega_j^{-1}\beta_{X_j}\tau_j + \theta_0\Omega_j^{-2}\sigma^2_{X_j}\tau_j^2$, $J_{1j} = \Omega_j^{-1}\beta_{X_j}(e_{Y_j} - \theta_0 e_{X_j}) + \Omega_j^{-2}(e_{Y_j} - \theta_0 e_{X_j})(\sigma^2_{Y_j}e_{X_j} + \theta_0\sigma^2_{X_j}e_{Y_j})$, $J_{2j} = \Omega_j^{-2}(\sigma^2_{Y_j}e_{X_j} + 2\theta_0\sigma^2_{X_j}e_{Y_j} - \theta_0^2\sigma^2_{X_j}e_{X_j})\tau_j$.

By CS,

$$\sum_{j \in S_0 \cup S} \Omega_j^{-2}\sigma^2_{X_j}\tau_j^2 = \Theta(n\|\tau\|_2^2) = O(1).$$

Also,

$$\begin{aligned} Var\Big(\sum_{j \in S_0 \cup S} J_{2j}\Big) &= \sum_{j \in S_0 \cup S} \Omega_j^{-4}\tau_j^2\sigma^2_{X_j}\left[(\sigma^2_{Y_j} - \theta_0^2\sigma^2_{X_j})^2 + 4\theta_0^2\sigma^2_{X_j}\sigma^2_{Y_j}\right] \\ &= \sum_{j \in S_0 \cup S} \Omega_j^{-2}\tau_j^2\sigma^2_{X_j} \\ &= O(1), \end{aligned}$$

where the last equality follows by $n\|\tau\|_2^2 = O(1)$ by Assumption 4. Therefore, by CH and $E[J_{2j}] = 0$ for all j, $\sum_{j \in S_0 \cup S} J_{2j} = O_P(1)$.

Then, since $\eta_C + \eta_S + \varsigma_C + \varsigma_S = \Theta(n\|\beta_X\|_2^2 + p)$,

$$\frac{1}{\sqrt{\eta_C + \eta_S + \varsigma_C + \varsigma_S}} \sum_{j \in S_0 \cup S} \left[\psi_j(\theta_0) - \Omega_j^{-1}\beta_{X_j}\tau_j\right] = \Big(\frac{1}{\sqrt{\eta_C + \eta_S + \varsigma_C + \varsigma_S}} \sum_{j \in S_0 \cup S} J_{1j}\Big) + o_P(1).$$

By identical arguments used in Zhao et al. (2020), $(\eta_C + \eta_S + \varsigma_C + \varsigma_S)^{-\frac{1}{2}} \sum_{j \in S_0 \cup S} J_{1j} \xrightarrow{D} N(0,1)$. The result then follows by Slutsky's lemma. □

Lemma S.4. Under Assumptions 1-4, for any $\bar{\theta} \xrightarrow{P} \theta_0$, $\sum_{j \in S_0 \cup S} \nabla_{\theta\theta}\psi_j(\bar{\theta})/(\eta_C + \eta_S) = O_P(1)$ as $n, p \to \infty$.

Proof. Note that $\nabla_\theta\Omega_j(\theta) = 2\theta\sigma^2_{X_j}$. For any θ, we can write

$$\begin{aligned} \nabla_{\theta\theta}\psi_j(\theta) = &-2\hat{\beta}_{X_j}\hat{\beta}_{Y_j}\sigma^2_{X_j}\Omega_j(\theta)^{-2} + 2\hat{\beta}_{X_j}(\hat{\beta}_{X_j}\sigma^2_{Y_j} + \theta\hat{\beta}_{Y_j}\sigma^2_{X_j})(\nabla_\theta\Omega_j(\theta))\Omega_j(\theta)^{-3} \\ &-2\hat{\beta}_{Y_j}\sigma^2_{X_j}(\hat{\beta}_{Y_j} - \theta\hat{\beta}_{X_j})(\nabla_\theta\Omega_j(\theta))\Omega_j(\theta)^{-3} \\ &-4\sigma^2_{X_j}(\hat{\beta}_{Y_j} - \theta\hat{\beta}_{X_j})(\hat{\beta}_{X_j}\sigma^2_{Y_j} + \theta\hat{\beta}_{Y_j}\sigma^2_{X_j})\Omega_j(\theta)^{-3} \\ &+4\theta\sigma^2_{X_j}\hat{\beta}_{X_j}(\hat{\beta}_{X_j}\sigma^2_{Y_j} + \theta\hat{\beta}_{Y_j}\sigma^2_{X_j})\Omega_j(\theta)^{-3} - 4\theta\sigma^4_{X_j}\hat{\beta}_{Y_j}(\hat{\beta}_{Y_j} - \theta\hat{\beta}_{X_j})\Omega_j(\theta)^{-3} \\ &+12\theta\sigma^2_{X_j}(\hat{\beta}_{Y_j} - \theta\hat{\beta}_{X_j})(\hat{\beta}_{X_j}\sigma^2_{Y_j} + \theta\hat{\beta}_{Y_j}\sigma^2_{X_j})(\nabla_\theta\Omega_j(\theta))\Omega_j(\theta)^{-4} \\ \equiv &\sum_{l=1}^{8} H_{lj}(\theta). \end{aligned}$$

We can expand $H_{1j}(\theta)$ so that

$$\begin{aligned} \sum_{j \in S_0 \cup S} H_{1j}(\theta) &= -\sum_{j \in S_0 \cup S} (\beta_{X_j} + e_{X_j})(\theta_0\beta_{X_j} + e_{Y_j} + \tau_j)\sigma^2_{X_j}\Omega_j(\theta)^{-2} \\ &= -\theta_0 \sum_{j \in S_0 \cup S} \sigma^2_{X_j}\Omega_j(\theta)^{-2}\beta^2_{X_j} - \sum_{j \in S_0 \cup S} \sigma^2_{X_j}\Omega_j(\theta)^{-2}\beta_{X_j}e_{Y_j} - \sum_{j \in S_0 \cup S} \sigma^2_{X_j}\Omega_j(\theta)^{-2}\beta_{X_j}\tau_j \end{aligned}$$

$$-\theta_0 \sum_{j\in S_0\cup S} \sigma_{X_j}^2 \Omega_j(\theta)^{-2}\beta_{X_j} e_{X_j} - \sum_{j\in S_0\cup S} \sigma_{X_j}^2 \Omega_j(\theta)^{-2} e_{X_j} e_{Y_j} - \sum_{j\in S_0\cup S} \sigma_{X_j}^2 \Omega_j(\theta)^{-2} e_{X_j}\tau_j.$$

For the first term on the right hand side, note that $\sum_{j\in S_0\cup S} \sigma_{X_j}^2 \Omega_j(\theta)^{-2}\beta_{X_j}^2 = \Theta(n\|\beta_X\|_2^2)$ since $\Omega_j(\theta) = \Theta(n^{-1})$. For the second term,

$$Var\Big(\sum_{j\in S_0\cup S} \sigma_{X_j}^2 \Omega_j(\theta)^{-2}\beta_{X_j} e_{Y_j}\Big) = \sum_{j\in S_0\cup S} \sigma_{X_j}^4 \sigma_{Y_j}^2 \Omega_j(\theta)^{-4}\beta_{X_j}^2 = \Theta(n\|\beta_X\|_2^2),$$

so that by CH, $\sum_{j\in S_0\cup S} \sigma_{X_j}^2 \Omega_j(\theta)^{-2}\beta_{X_j} e_{Y_j} = O(\sqrt{n}\|\beta_X\|_2)$.

Similarly,

$$\Big|\sum_{j\in S_0\cup S} \sigma_{X_j}^2 \Omega_j(\theta)^{-2}\beta_{X_j}\tau_j\Big| \le \Theta(n\|\beta_X\|_2\|\tau\|_2) = O(\sqrt{n}\|\beta_X\|_2).$$

For the fifth term on the right hand side,

$$Var\Big(\sum_{j\in S_0\cup S} \sigma_{X_j}^2 \Omega_j(\theta)^{-2} e_{X_j} e_{Y_j}\Big) = \Theta\Big(n^2 \sum_{j\in S_0\cup S} \sigma_{X_j}^2 \sigma_{Y_j}^2\Big) = \Theta(p),$$

so that $\sum_{j\in S_0\cup S} \sigma_{X_j}^2 \Omega_j(\theta)^{-2} e_{X_j} e_{Y_j} = O(\sqrt{p})$ by CH.

Using similar arguments for the remaining terms of $\sum_{j\in S_0\cup S} H_{1j}(\theta)$, and for $\sum_{j\in S_0\cup S} H_{lj}(\theta)$, $l = 2,\ldots,8$, we have that

$$\sum_{l=1}^{8} \sum_{j\in S_0\cup S} H_{lj}(\theta) = O_P(n\|\beta_X\|_2^2 + p)$$

as $n,p\to\infty$, which leads to the required result since $\eta_C + \eta_S = \Theta(n\|\beta_X\|_2^2)$ and $p/(n\|\beta_X\|_2^2) = O(1)$ by Assumption 3. □

Proof of Theorem 1 (Asymptotic distribution of $\hat\theta_S$).

The proof strategy follows Zhao et al. (2020)'s Proof of Theorem 3.2, but with Lemmas S.2-S.4 involving additional steps required to control the non-negligible asymptotic bias term from our model.

Let $\psi_j(\theta) = (\hat\beta_{Y_j} - \theta\hat\beta_{X_j})(\hat\beta_{X_j}\sigma_{Y_j}^2 + \theta\hat\beta_{Y_j}\sigma_{X_j}^2)(\sigma_{Y_j}^2 + \theta^2\sigma_{X_j}^2)^{-2}$. Given consistency of $\hat\theta_S$, a second-order Taylor expansion of the first-order condition $\sum_{j\in S_0\cup S}\psi_j(\hat\theta_S) = 0$ around $\hat\theta_S = \theta_0$ implies that there exists $\bar\theta$ on the line segment joining $\hat\theta_S$ and θ_0 such that

$$\frac{\eta_C+\eta_S}{\sqrt{\eta_C+\eta_S+\varsigma_C+\varsigma_S}}(\hat\theta_S-\theta_0) = -\Big(\frac{1}{\eta_C+\eta_S}\sum_{j\in S_0\cup S}\nabla_\theta\psi_j(\theta_0) + o_P\Big(\frac{1}{\eta_C+\eta_S}\sum_{j\in S_0\cup S}\nabla_{\theta\theta}\psi_j(\bar\theta)\Big)\Big)^{-1} \times \frac{1}{\sqrt{\eta_C+\eta_S+\varsigma_C+\varsigma_S}}\sum_{j\in S_0\cup S}\psi_j(\theta_0).$$

The result then follows by Slutsky's lemma, and Lemmas S.2-S.4, which show, as $n,p\to\infty$:

(i) $\sum_{j\in S_0\cup S}\nabla_\theta\psi_j(\theta_0)/(\eta_C+\eta_S) \xrightarrow{P} -1$;

(ii) $\sum_{j\in S_0\cup S}\psi_j(\theta_0)/\sqrt{\eta_C+\eta_S+\varsigma_C+\varsigma_S} \xrightarrow{D} N(b_S/\sqrt{\eta_C+\eta_S+\varsigma_C+\varsigma_S}, 1)$;

(iii) for any $\bar\theta \xrightarrow{P} \theta_0$, $\sum_{j\in S_0\cup S}\nabla_{\theta\theta}\psi_j(\bar\theta)/(\eta_C+\eta_S) = O_P(1)$. □

Proof of Theorem 2 (Asymptotic distribution of the bias $\hat b_S$).

We show that $\hat{V}_B^{-\frac{1}{2}}(\hat{b}_S - b_S) \xrightarrow{D} N(0,1)$ as $n, p \to \infty$, where $\hat{V}_B = \hat{\eta}_S + \hat{\varsigma}_S + \hat{\xi}_S + (\hat{\eta}_S^2/\hat{\eta}_C^2)(\hat{\eta}_C + \hat{\varsigma}_C)$. The result of Theorem 2 then follows by $(\hat{\eta}_C + \hat{\eta}_S)/(\eta_C + \eta_S) \xrightarrow{P} 1$ shown below in Proof of Lemma S.5, and Slutsky's lemma.

Let $\hat{B}_j(\theta) = \hat{\Omega}_j^{-1}\hat{\beta}_{X_j}(\hat{\beta}_{Y_j} - \theta\hat{\beta}_{X_j}) + \theta\hat{\Omega}_j^{-1}\sigma_{X_j}^2$. Note that $\hat{\Omega}_j - \Omega_j = (\hat{\theta} - \theta_0)(\hat{\theta} + \theta_0)\sigma_{X_j}^2 = \Theta(n^{-1}|\hat{\theta} - \theta_0|)$ implies that $\hat{\Omega}_j^{-1} - \Omega_j^{-1} = \hat{\Omega}_j^{-1}(\Omega_j - \hat{\Omega}_j)\Omega_j^{-1} = \Theta(n|\hat{\theta} - \theta_0|)$.

We can write

$$\sum_{j\in S}\big[\hat{B}_j(\theta_0) - \Omega_j^{-1}\beta_{X_j}\tau_j\big] = \sum_{j\in S}\Big[\Omega_j^{-1}\beta_{X_j}(e_{Y_j} - \theta_0 e_{X_j}) + \Omega_j^{-1}e_{X_j}e_{Y_j} - \theta_0\Omega_j^{-1}(e_{X_j}^2 - \sigma_{X_j}^2)$$
$$+(\hat{\Omega}_j^{-1} - \Omega_j^{-1})\beta_{X_j}(e_{Y_j} - \theta_0 e_{X_j}) + (\hat{\Omega}_j^{-1} - \Omega_j^{-1})e_{X_j}e_{Y_j}$$
$$-\theta_0(\hat{\Omega}_j^{-1} - \Omega_j^{-1})(e_{X_j}^2 - \sigma_{X_j}^2) + (\hat{\Omega}_j^{-1} - \Omega_j^{-1})\beta_{X_j}\tau_j$$
$$+\Omega_j^{-1}\tau_j e_{X_j} + (\hat{\Omega}_j^{-1} - \Omega_j^{-1})\tau_j e_{X_j}\Big].$$

Using $\hat{\Omega}_j^{-1} - \Omega_j^{-1} = \Theta(n|\hat{\theta} - \theta_0|)$, note that $\big|\sum_{j\in S}(\hat{\Omega}_j^{-1} - \Omega_j^{-1})\beta_{X_j}(e_{Y_j} - \theta_0 e_{X_j})\big| = O_P(\sqrt{n}|\hat{\theta} - \theta_0|\|\beta_X\|_2) = o_P(\sqrt{n}\|\beta_X\|_2)$ by CS, CH, and consistency of $\hat{\theta}_C$ for θ_0. Similarly, the last five terms on the right hand side are $o_P(\sqrt{n}\|\beta_X\|_2 + \sqrt{p})$. Therefore, for $\bar{B}_j(\theta_0) = \Omega_j^{-1}\beta_{X_j}(e_{Y_j} - \theta_0 e_{X_j}) + \Omega_j^{-1}e_{X_j}e_{Y_j} - \theta_0\Omega_j^{-1}(e_{X_j}^2 - \sigma_{X_j}^2)$,

$$\sum_{j\in S}\big[\hat{B}_j(\theta_0) - \Omega_j^{-1}\beta_{X_j}\tau_j\big] = \sum_{j\in S}\bar{B}_j(\theta_0) + o_P(\sqrt{n}\|\beta_X\|_2 + \sqrt{p}).$$

By CH, $|e_{X_j}| = O_P(n^{-\frac{1}{2}})$ and $|e_{Y_j}| = O_P(n^{-\frac{1}{2}})$, so that $E[|\bar{B}_j(\theta_0)|^3] = O(n^{\frac{3}{2}}|\beta_{X_j}|^3) + O(n|\beta_{X_j}|^2) + O(\sqrt{n}|\beta_{X_j}|) + O(1)$, and

$$\sum_{j\in S}E\big[|\bar{B}_j(\theta_0)|^3\big] = O(n^{\frac{3}{2}}\|\beta_X\|_3^3) + O(n\|\beta_X\|_2^2) + O(\sqrt{n}\|\beta_X\|_1) + O(p)$$
$$= O(n^{\frac{3}{2}}\|\beta_X\|_3^3) + O(n\|\beta_X\|_2^2) + O(p),$$

where the last equality follows by $\sqrt{n}\|\beta_X\|_1 \le \sqrt{p}\sqrt{n}\|\beta_X\|_2 \le (n\|\beta_X\|_2^2 + p)/2$.

Let $\xi_S = 2\theta_0^2\sum_{j\in S}\Omega_j^{-2}\sigma_{X_j}^4$. The variance of $\sum_{j\in S}\bar{B}_j(\theta_0)$ is given by

$$Var\Big(\sum_{j\in S}\bar{B}_j(\theta_0)\Big) = \eta_S + \varsigma_S + \xi_S,$$

where $\eta_S + \varsigma_S = \Theta(n\|\beta_X\|_2^2 + p)$ and $\xi_S = \Theta(p)$. Therefore, the following Lyapanov condition holds,

$$\frac{1}{(\eta_S + \varsigma_S + \xi_S)^{\frac{3}{2}}}\sum_{j\in S}E\big[|\bar{B}_j(\theta_0)|^3\big] = O\Big(\frac{\|\beta_X\|_3}{\|\beta_X\|_2}\Big) + O\Big(\frac{1}{\sqrt{n}\|\beta_X\|_2}\Big) + O\Big(\frac{1}{\sqrt{p}}\Big)$$
$$= o(1),$$

by $\|\beta_X\|_3/\|\beta_X\|_2 \to 0$ in Assumption 3. Thus, by Lyapanov's CLT,

$$\frac{1}{\sqrt{\eta_S + \varsigma_S + \xi_S}}\sum_{j\in S}\bar{B}_j(\theta_0) \xrightarrow{D} N(0,1).$$

Note that $\hat{B}_j(\hat{\theta}_C) - \hat{B}_j(\theta_0) = -(\hat{\theta}_C - \theta_0)\hat{\Omega}_j^{-1}(\hat{\beta}_{X_j}^2 - \sigma_{X_j}^2)$, and

$$\sum_{j\in S}\hat{\Omega}_j^{-1}(\hat{\beta}_{X_j}^2 - \sigma_{X_j}^2) = \sum_{j\in S}\Big[\Omega_j^{-1}\beta_{X_j}^2 + (\hat{\Omega}_j^{-1} - \Omega_j^{-1})\beta_{X_j}^2 + 2\Omega_j^{-1}\beta_{X_j}e_{X_j} + 2(\hat{\Omega}_j^{-1} - \Omega_j^{-1})\beta_{X_j}e_{X_j}$$

$$+\Omega_j^{-1}(e_{X_j}^2-\sigma_{X_j}^2)+(\hat{\Omega}_j^{-1}-\Omega_j^{-1})(e_{X_j}^2-\sigma_{X_j}^2)\Big].$$

By similar arguments used above, and since $p/n\|\beta_X\|_2^2=O(1)$ by Assumption 3, the last five terms on the right hand side are $o_P(n\|\beta_X\|_2^2)$.

Therefore, since $\hat{\theta}_C-\theta_0=O_P(1/\sqrt{n}\|\beta_{X,0}\|_2)+O(\sqrt{p}/n\|\beta_{X,0}\|_2^2)$, by the above results,

$$\sum_{j\in S}\big[\hat{B}_j(\hat{\theta}_C)-\Omega_j^{-1}\beta_{X_j}\tau_j\big]=\sum_{j\in S}\bar{B}_j(\theta_0)-\eta_S(\hat{\theta}_C-\theta_0)+o_P(\sqrt{n}\|\beta_X\|_2+\sqrt{p}).$$

Let $V_B=\eta_S+\varsigma_S+\xi_S+(\eta_S^2/\eta_C^2)(\eta_C+\varsigma_C)$. Note that $V_B=\Theta(n\|\beta_X\|_2^2+p)$ so that

$$V_B^{-\frac{1}{2}}\sum_{j\in S}\big[\hat{B}_j(\hat{\theta}_C)-\Omega_j^{-1}\beta_{X_j}\tau_j\big]=V_B^{-\frac{1}{2}}\Big(\sum_{j\in S}\bar{B}_j(\theta_0)-\eta_S(\hat{\theta}_C-\theta_0)\Big)+o_P(1).$$

It can be shown that the Core estimator has the first order expansion

$$\hat{\theta}_C-\theta_0=\frac{1}{\eta_C}\sum_{j\in S_0}J_{1j}+o_P\Big(\frac{\sqrt{\eta_C+\varsigma_C}}{\eta_C}\Big),$$

so that

$$V_B^{-\frac{1}{2}}\sum_{j\in S}\big[\hat{B}_j(\hat{\theta}_C)-\Omega_j^{-1}\beta_{X_j}\tau_j\big]=V_B^{-\frac{1}{2}}\Big(\sum_{j\in S}\bar{B}_j(\theta_0)-\frac{\eta_S}{\eta_C}\sum_{j\in S_0}J_{1j}\Big)+o_P(1),$$

as $o_P(V_B^{-\frac{1}{2}}(\eta_S/\eta_C^2)\sqrt{\eta_C+\zeta_C})=o_P(1)$. From the above arguments, and noting that $\sum_{j\in S}\bar{B}_j(\theta_0)$ and $\sum_{j\in S_0}J_{1j}$ are mutually independent, the result follows by Slutsky's lemma. □

Lemma S.5 (Consistent variance estimation).

Let $\hat{\Omega}_j=\sigma_{Y_j}^2+\hat{\theta}_C^2\sigma_{X_j}^2$, $\hat{V}_B=\hat{\eta}_S+\hat{\varsigma}_S+\hat{\xi}_S+(\hat{\eta}_S^2/\hat{\eta}_C^2)(\hat{\eta}_C+\hat{\varsigma}_C)$, where $\hat{\xi}_S=2\hat{\theta}_C^2\sum_{j\in S}\hat{\Omega}_j^{-2}\sigma_{X_j}^4$. We show that, under Assumptions 1-4, as $n,p\to\infty$,

(i) $(\eta_C+\eta_S)^2(\hat{\eta}_C+\hat{\eta}_S+\hat{\varsigma}_C+\hat{\varsigma}_S)/(\hat{\eta}_C+\hat{\eta}_S)^2(\eta_C+\eta_S+\varsigma_C+\varsigma_S)\xrightarrow{P}1$;
(ii) $\hat{V}_B/V_B\xrightarrow{P}1$.

Proof.

Part (i)

First, note that $\hat{\Omega}_j-\Omega_j=2\sigma_{X_j}^2(\hat{\theta}_S-\theta_0)=\Theta(|\hat{\theta}_S-\theta_0|/n)$, so that $\hat{\Omega}_j^{-1}-\Omega_j^{-1}=\hat{\Omega}_j^{-1}(\Omega_j-\hat{\Omega}_j)\Omega_j^{-1}=\Theta(n|\hat{\theta}_S-\theta_0|)$.

Then,

$$\begin{aligned}\hat{\eta}_C+\hat{\eta}_S-\eta_C-\eta_S&=2\sum_{j\in S_0\cup S}\Omega_j^{-1}\beta_{X_j}e_{X_j}+\sum_{j\in S_0\cup S}\Omega_j^{-1}(e_{X_j}^2-\sigma_{X_j}^2)+\sum_{j\in S_0\cup S}(\hat{\Omega}_j^{-1}-\Omega_j^{-1})\beta_{X_j}^2\\&\quad+2\sum_{j\in S_0\cup S}(\hat{\Omega}_j^{-1}-\Omega_j^{-1})\beta_{X_j}e_{X_j}+\sum_{j\in S_0\cup S}(\hat{\Omega}_j^{-1}-\Omega_j^{-1})(e_{X_j}^2-\sigma_{X_j}^2)\\&=O_P(\sqrt{n}\|\beta_X\|_2)+O_P(\sqrt{p})+o_P(n\|\beta_X\|_2^2)\\&=o_P(n\|\beta_X\|_2^2)+O_P(\sqrt{p}),\end{aligned}$$

by similar arguments used in the proof of Theorem 2. By T, since $\eta_C+\eta_S=\Theta(n\|\beta_X\|_2^2)$ and $p/n^2\|\beta_X\|_2^4\to0$, we have $(\hat{\eta}_C+\hat{\eta}_S)^{-1}=O_P(1/n\|\beta_X\|_2^2)$.

Therefore,

$$\frac{(\eta_C+\eta_S)^2}{(\hat\eta_C+\hat\eta_S)^2}-1 = 2(\eta_C+\eta_S+\hat\eta_C+\hat\eta_S)(\eta_C+\eta_S-\hat\eta_C-\hat\eta_S)/(\hat\eta_C+\hat\eta_S)^2$$
$$= O_P(n\|\beta_X\|_2^2)[o_P(n\|\beta_X\|_2^2)+O_P(\sqrt{p})]O_P(1/n^2\|\beta_X\|_2^4)$$
$$= o_P(1)+O_P(\sqrt{p}/n\|\beta_X\|_2^2)$$
$$= o_P(1),$$

where the last line follows from $p/n^2\|\beta_X\|_2^4 \to 0$ as $n,p\to\infty$, which is implied by Assumption 3.

Similarly, noting that $\hat\Omega_j^{-2}-\Omega_j^{-2} = \hat\Omega_j^{-2}\Omega_j^{-2}(\Omega_j+\hat\Omega_j)(\Omega_j-\hat\Omega_j) = \Theta(n^2|\hat\theta_S-\theta_0|)$, we have

$$\hat\varsigma_C+\hat\varsigma_S-\varsigma_C-\varsigma_S = \sum_{j\in S_0\cup S}(\hat\Omega_j^{-2}-\Omega_j^{-2})\sigma_{X_j}^2\sigma_{Y_j}^2$$
$$= \Theta(p|\hat\theta_S-\theta_0|)$$
$$= o_P(p).$$

Using the above results,

$$\frac{(\eta_C+\eta_S)^2}{(\hat\eta_C+\hat\eta_S)^2}\cdot\frac{\hat\eta_C+\hat\eta_S+\hat\varsigma_C+\hat\varsigma_S}{\eta_C+\eta_S+\varsigma_C+\varsigma_S} = (1+o_P(1))\Big(1+\frac{\hat\eta_C+\hat\eta_S-\eta_C-\eta_S}{\eta_C+\eta_S+\varsigma_C+\varsigma_S}+\frac{\hat\varsigma_C+\hat\varsigma_S-\varsigma_C-\varsigma_S}{\eta_C+\eta_S+\varsigma_C+\varsigma_S}\Big)$$
$$= (1+o_P(1))\Big(1+\frac{o_P(n\|\beta_X\|_2^2)+O_P(\sqrt{p})+o_P(p)}{\Theta(n\|\beta_X\|_2^2)+\Theta(p)}\Big)$$
$$= (1+o_P(1))^2$$
$$= 1+o_P(1).$$

Part (ii)

First, note that

$$\hat\xi_S-\xi_S = 2(\hat\theta_S+\theta_0)(\hat\theta_S-\theta_0)\sum_{j\in S}\Omega_j^{-2}\sigma_{X_j}^4+2(\hat\theta_S+\theta_0)(\hat\theta_S-\theta_0)\sum_{j\in S}(\hat\Omega_j^{-2}-\Omega_j^{-2})\sigma_{X_j}^4$$
$$+2\theta_0^2\sum_{j\in S}(\hat\Omega_j^{-2}-\Omega_j^{-2})\sigma_{X_j}^4$$
$$= \Theta(p|\hat\theta_S-\theta_0|)+\Theta(p|\hat\theta_S-\theta_0|^2)$$
$$= o_P(p).$$

Therefore,

$$\hat V_B-V_B = (\hat\eta_S-\eta_S)+(\hat\varsigma_S-\varsigma_S)+(\hat\xi_S-\xi_S)+(\hat\eta_S-\eta_S)(\hat\eta_S+\eta_S)\frac{(\hat\eta_C+\hat\zeta_V)}{\hat\eta_C^2}$$
$$+\eta_S^2\Big(\frac{\hat\eta_C+\hat\zeta_C}{\hat\eta_C^2}-\frac{\eta_C+\zeta_C}{\eta_C^2}\Big)$$
$$= o_P(n\|\beta_X\|_2^2)+O_P(\sqrt{p})+o_P(p)+\big(o_P(n\|\beta_X\|_2^2)+O_P(\sqrt{p})\big)O_P(n\|\beta_X\|_2^2)\big[O_P(1/n\|\beta_X\|_2^2)$$
$$+o_P(1)\big]+O(n^2\|\beta_X\|_2^4)\big(o_P(1/n\|\beta_X\|_2^2)+o_P(p/n^2\|\beta_X\|_2^4)\big)$$
$$= o_P(n\|\beta_X\|_2^2)+o_P(p)+o_P(p^2/n\|\beta_X\|_2^2)$$

where the second equality follows by (i) and T, since $\hat\eta_C^{-2}(\hat\eta_C+\hat\varsigma_C) = O_P(\eta_C^{-2}(\eta_C+\varsigma_C)) = O_P(1/n\|\beta_X\|_2^2)+O_P(p/n^2\|\beta_X\|_2^4)$, and $\hat\eta_C^{-2}(\hat\eta_C+\hat\varsigma_C)-\eta_C^{-2}(\eta_C+\varsigma_C) = o_P(\eta_C^{-2}(\eta_C+\varsigma_C)) = o_P(1/n\|\beta_X\|_2^2)+o_P(p/n^2\|\beta_X\|_2^4)$.

Therefore, since $V_B = \Theta(n\|\beta_X\|_2^2) + \Theta(p)$,

$$\frac{\hat{V}_B - V_B}{V_B} = o_P(1) + o_P\Big(\frac{p}{n\|\beta_X\|_2^2}\Big)$$
$$= o_P(1),$$

as $p/n\|\beta_X\|_2^2 = O(1)$. Finally, we note $\hat{V}_B/V_B = 1 + (\hat{V}_B - V_B)/V_B = 1 + o_P(1)$. □

Proof of Theorem 3 – Part I (Convergence in distribution of effect and bias estimates).

As shown in the proofs of Theorem 1 and 2, for any S, ignoring $o_P(1/\sqrt{n}\|\beta_X\|_2)$ and $o_P(\sqrt{p}/n\|\beta_X\|_2^2)$ terms,

$$\hat{\theta}_C - \theta_0 = \frac{1}{\eta_C}\sum_{j\in S_0} J_{1j}$$

$$\hat{\theta}_{S_k} - \theta_0 - \frac{b_{S_k}}{\eta_C + \eta_{S_k}} = \frac{1}{\eta_C + \eta_{S_k}}\sum_{j\in S_0\cup S_k} J_{1j}$$

$$\frac{\hat{b}_{S_k} - b_{S_k}}{\eta_C + \eta_{S_k}} = \frac{1}{\eta_C + \eta_{S_k}}\sum_{j\in S_k}\bar{B}_j - \frac{\eta_{S_k}}{\eta_C(\eta_C + \eta_{S_k})}\sum_{j\in S_0} J_{1j}$$

where $J_{1j} = \Omega_j^{-1}\beta_{X_j}(e_{Y_j} - \theta_0 e_{X_j}) + \Omega_j^{-2}(e_{Y_j} - \theta_0 e_{X_j})(\sigma_{Y_j}^2 e_{X_j} + \theta_0\sigma_{X_j}^2 e_{Y_j})$, and $\bar{B}_j = \Omega_j^{-1}\beta_{X_j}(e_{Y_j} - \theta_0 e_{X_j}) + \Omega_j^{-1}e_{X_j}e_{Y_j} - \theta_0\Omega_j^{-1}(e_{X_j}^2 - \sigma_{X_j}^2)$.

We can partition the K additional instrument sets into $L \le 2^K - 1$ *distinct* sets which span the additional instrument sets $S_1, ..., S_K$. For example, for $K = 3$, each instrument must belong to one, and only one, of the following sets: $M_1 = S_1 \cap S_2 \cap S_3$, $M_2 = S_1 \cap S_2 \cap S_3^C$, $M_3 = S_1 \cap S_2^C \cap S_3$, $M_4 = S_1^C \cap S_2 \cap S_3$, $M_5 = S_1 \cap S_2^C \cap S_3^C$, $M_6 = S_1^C \cap S_2 \cap S_3^C$, and $M_7 = S_1^C \cap S_2^C \cap S_3$. Then, for each $j \in [3]$, we can construct selection indicators $\alpha_\ell \in \{0, 1\}$, $\ell \in [7]$, such that $S_j = \bigcup_{\ell=1}^{7}\alpha_\ell M_\ell$.

For $L \le 2^{|K|} - 1$, let $M_1, ..., M_L$ be distinct sets of the additional instruments which span the additional instrument sets $S_1, ..., S_K$.

We can therefore write

$$\begin{pmatrix} \hat{\theta}_C - \theta_0 \\ \hat{\theta}_{S_1} - \theta_0 - \frac{b_{S_1}}{\eta_C + \eta_{S_1}} \\ \vdots \\ \hat{\theta}_{S_K} - \theta_0 - \frac{b_{S_K}}{\eta_C + \eta_{S_K}} \\ \frac{\hat{b}_{S_1} - b_{S_1}}{\eta_C + \eta_{S_1}} \\ \vdots \\ \frac{\hat{b}_{S_K} - b_{S_K}}{\eta_C + \eta_{S_K}} \end{pmatrix} = \pi_C\left(\textstyle\sum_{j\in S_0} J_{1j}\right) + \sum_{\ell=1}^{L}\pi_{M_\ell}\left(\textstyle\sum_{j\in M_\ell}\mu_j\right),$$

where

$$\mu_j = \begin{pmatrix} J_{1j} \\ H_j \end{pmatrix}, \ \pi_C = \begin{pmatrix} \frac{1}{\eta_C} \\ \frac{1}{\eta_C+\eta_{S_1}} \\ \vdots \\ \frac{1}{\eta_C+\eta_{S_K}} \\ -\frac{\eta_{S_1}}{\eta_C(\eta_C+\eta_{S_1})} \\ \vdots \\ -\frac{\eta_{S_K}}{\eta_C(\eta_C+\eta_{S_K})} \end{pmatrix}, \ \pi_{M_\ell} = \begin{pmatrix} 0 & 0 \\ \frac{\mathbb{I}\{M_\ell \subseteq S_1\}}{\eta_C+\eta_{S_1}} & 0 \\ \vdots & \vdots \\ \frac{\mathbb{I}\{M_\ell \subseteq S_K\}}{\eta_C+\eta_{S_K}} & 0 \\ \cdot & \frac{\mathbb{I}\{M_\ell \subseteq S_1\}}{\eta_C+\eta_{S_1}} \\ \vdots & \vdots \\ 0 & \frac{\mathbb{I}\{M_\ell \subseteq S_K\}}{\eta_C+\eta_{S_K}} \end{pmatrix} \begin{pmatrix} 1 & 0 \\ 1 & -1 \end{pmatrix}, (\ell = 1, .., L).$$

and $H_j = \Omega_j^{-2}\theta_0(\sigma^2_{X_j}e^2_{Y_j} - \sigma^2_{Y_j}e^2_{X_j} - 2\theta_0\sigma^2_{X_j}e_{X_j}e_{Y_j}) + \theta_0\Omega_j^{-1}(e^2_{X_j} - \sigma^2_{X_j})$. For any set M_ℓ, we will show that

$$\text{(1)} \qquad \sum_{j\in M_\ell} \mu_j \overset{a}{\sim} N\left(\begin{bmatrix} 0 \\ 0 \end{bmatrix}, \begin{bmatrix} \eta_{M_\ell} + \varsigma_{M_\ell} & 0 \\ 0 & \xi_{M_\ell} \end{bmatrix}\right).$$

Also, as in the Proof of Lemma S.3, we have

$$\sum_{j\in S_0} J_{1j} \overset{a}{\sim} N(0, \eta_C + \varsigma_C).$$

Then, since (i) the random components in J_{1j} and μ_j are functions of the error terms e_{X_j} and e_{Y_j}; (ii) for any $j \neq k$, e_{X_j} and e_{X_k} are jointly normal and uncorrelated, and hence mutually independent (likewise for e_{Y_j} and e_{X_j}), we have that $\sum_{j\in S_0} J_{1j}$, $\sum_{j\in M_1}\mu_j$, ..., $\sum_{j\in M_{K_0}}\mu_j$ are mutually independent sums. Therefore,

$$\pi_C\left(\textstyle\sum_{j\in S_0} J_{1j}\right) + \sum_{\ell=1}^{L} \pi_{M_\ell}\left(\textstyle\sum_{j\in M_\ell}\mu_j\right) \overset{a}{\sim} N\left(\underset{((2K+1)\times 1)}{0}, R + W\right),$$

where $R = \pi_C(\eta_C + \zeta_C)\pi_C'$ and $W = \sum_{\ell=1}^{L}\pi_{M_\ell}\begin{bmatrix} \eta_{M_\ell} + \varsigma_{M_\ell} & 0 \\ 0 & \xi_{M_\ell} \end{bmatrix}\pi'_{M_\ell}$.

Some straight-forward calculations show that

$$R = \begin{bmatrix} \underset{(1\times 1)}{R_{11}} & \underset{(1\times K)}{R'_{21}} & \underset{(1\times K)}{R'_{31}} \\ \underset{(K\times 1)}{R_{21}} & \underset{(K\times K)}{R_{22}} & \underset{(K\times K)}{R'_{32}} \\ \underset{(K\times 1)}{R_{31}} & \underset{(K\times K)}{R_{32}} & \underset{(K\times K)}{R_{33}} \end{bmatrix},$$

where $R_{11} = \eta_C^{-2}(\eta_C + \varsigma_C)$, the k-th element of R_{21} is given by $R_{21}^{(k)} = \eta_C^{-1}(\eta_C + \eta_{S_k})^{-1}(\eta_C + \varsigma_C)$, the k-th element of R_{31} is given by $R_{31}^{(k)} = -\eta_C^{-2}(\eta_C + \eta_{S_k})^{-1}\eta_{S_k}(\eta_C + \varsigma_C)$, the (k,l)-th element of R_{22} is given by $R_{22}^{(k,l)} = (\eta_C + \eta_{S_k})^{-1}(\eta_C + \eta_{S_l})^{-1}(\eta_C + \varsigma_C)$, the (k,l)-th element of R_{32} is given by $R_{32}^{(k,l)} = -\eta_C^{-1}\eta_{S_k}(\eta_C + \eta_{S_k})^{-1}(\eta_C + \eta_{S_l})^{-1}(\eta_C + \varsigma_C)$, and the (k,l)-th element of R_{33} is given by $R_{33}^{(k,l)} = \eta_C^{-2}(\eta_C + \eta_{S_k})^{-1}(\eta_C + \eta_{S_l})^{-1}\eta_{S_k}\eta_{S_k}(\eta_C + \varsigma_C)$, $(k, l = 1, ..., K)$.

Similarly, we have

$$W = \begin{bmatrix} \underset{(1\times 1)}{0} & \underset{(1\times K)}{0} & \underset{(1\times K)}{0} \\ \underset{(K\times 1)}{0} & \underset{(K\times K)}{W_1} & \underset{(K\times K)}{W_1} \\ \underset{(K\times 1)}{0} & \underset{(K\times K)}{W_1} & \underset{(K\times K)}{W_2} \end{bmatrix},$$

where the (k,l)-th element of W_1 is given by $W_1^{(k,l)} = (\eta_C + \eta_{S_k})^{-1}(\eta_C + \eta_{S_l})^{-1}(\eta_{S_k \cap S_l} + \varsigma_{S_k \cap S_l})$, and the (k,l)-th element of W_2 is given by $W_2^{(k,l)} = (\eta_C + \eta_{S_k})^{-1}(\eta_C + \eta_{S_l})^{-1}(\eta_{S_k \cap S_l} + \varsigma_{S_k \cap S_l} + \xi_{S_k \cap S_l})$, $(k,l = 1,...,K)$.

The expression for the covariance matrix in Theorem 3 is then $\Delta = R + W$.

Proof of Equation 1.

If $|M_\ell| = o(p)$, then the following asymptotic distribution result for $\sum_{j \in M_\ell} \mu_j$ still applies but variance components ς_{M_ℓ} and ξ_{M_ℓ} from Equation 4 would be negligible. Therefore, we focus on the case $|M_\ell| = O(p)$ for $\ell \in [L]$. We wish to apply the following multivariate Berry-Esseen result by Bentkus (2005). For a subset of instruments M_ℓ and the 2-dimensional vector of independent variables $\mu_1, ..., \mu_{|M_\ell|}$, let $\Delta_\ell = Var\big(\sum_{j \in M_\ell} \mu_j\big)$. Then for $U \sim N(0, \Delta_\ell)$, for any convex set S,

$$\Big|P\big(\sum_{j \in M_\ell} \mu_j \in S\big) - P(U \in S)\Big| \leq O\Big(\sum_{j \in M_\ell} E[\|\Delta_\ell^{-\frac{1}{2}} \mu_j\|_2^3]\Big).$$

Thus, joint convergence of effect and bias estimates follows by the following equations

$$\Delta_\ell = \Theta(n\|\beta_X\|_2^2 + p) \tag{2}$$

$$\sum_{j \in M_\ell} E[\|\mu_j\|_2^3] = O(n^{\frac{3}{2}}\|\beta_X\|_4^2\|\beta_X\|_2) + O(\sqrt{p}n\|\beta_X\|_4^2) + O(n\|\beta_X\|_2^2) + O(p) \tag{3}$$

since (2) implies $\Delta_\ell^{-\frac{1}{2}} = \Theta(1/\sqrt{n}\|\beta_X\|_2) + \Theta(1/\sqrt{p})$, and by (3),

$$\begin{aligned}
\sum_{j \in M_\ell} E[\|\Delta_\ell^{-\frac{1}{2}} \mu_j\|_2^3] &= \Delta_\ell^{-\frac{3}{2}} \sum_{j \in M_\ell} E[\|\mu_j\|_2^3] \\
&= O\Big(\frac{\|\beta_X\|_4^2}{\|\beta_X\|_2^2}\Big) + O\Big(\frac{\sqrt{p}}{\sqrt{n}\|\beta_X\|_2}\Big) O\Big(\frac{\|\beta_X\|_4^2}{\|\beta_X\|_2^2}\Big) + O\Big(\frac{1}{\sqrt{n}\|\beta_X\|_2}\Big) + O\Big(\frac{1}{\sqrt{p}}\Big) \\
&= o(1),
\end{aligned}$$

as $\|\beta_X\|_4/\|\beta_X\|_2 \to 0$ and $p/n\|\beta_X\|_2^2 = O(1)$ by Assumption 3.

To show (2), we calculate the covariance matrix $\Delta_\ell = Var(\sum_{j \in M_\ell} \mu_j)$ as

$$\begin{aligned}
\Delta_\ell &= \sum_{j \in M_\ell} Var(\mu_j) \\
&= \begin{bmatrix} \eta_{M_\ell} + \varsigma_{M_\ell} & 0 \\ 0 & \xi_{M_\ell} \end{bmatrix} \\
&= \Theta(n\|\beta_X\|_2^2 + p),
\end{aligned}$$

since $Var(J_{1j}) = \Omega_j^{-1}\beta_{X_j}^2 + \Omega_j^{-2}\sigma_{X_j}^2\sigma_{Y_j}^2$, $Cov(J_{1j}, H_j) = 0$, and $Var(H_j) = 2\theta_0^2\Omega_j^{-2}\sigma_{X_j}^4$.

To show (3), note that $\|\mu_j\|_2^2 = J_{1j}^2 + (J_{1j} - \bar{B}_j)^2 \leq 3J_{1j}^2 + 2\bar{B}_j^2$, and $\|\mu_j\|_2^4 \leq 9J_{1j}^4 + 12J_{1j}^2\bar{B}_j^2 + 4\bar{B}_j^4$. Thus, $E[\|\mu_j\|_2^2] = 3E[J_{1j}^2] + 2E[\bar{B}_j^2]$, and $E[\|\mu_j\|_2^4] \leq 15E[J_{1j}^4] + 10E[\bar{B}_{1j}^4]$.

Then,

$$\begin{aligned}
\sum_{j \in M_\ell} E[\|\mu_j\|_2^2] &= 3\sum_{j \in M_\ell} E[J_{1j}^2] + 2\sum_{j \in M_\ell} E[\bar{B}_j^2] \\
&= 5\eta_{M_\ell} + 5\varsigma_{M_\ell} + 2\xi_{M_\ell} \\
&= \Theta(n\|\beta_X\|_2^2 + p),
\end{aligned}$$

and

$$\sum_{j\in M_\ell} E[\|\mu_j\|_2^4] \le 15 \sum_{j\in M_\ell} E[J_{1j}^4] + 10 \sum_{j\in M_\ell} E[\bar{B}_j^4]$$

$$= O(n^2\|\beta_X\|_4^4) + O(n\|\beta_X\|_2^2) + O(p),$$

since by direct calculation, it can be shown that there exist constants C_l, $l \in [10]$ such that

$$\sum_{j\in M_\ell} E[J_{1j}^4] = C_1 \sum_{j\in M_\ell} \Omega_j^{-2}\beta_{X_j}^4 + C_2 \sum_{j\in M_\ell} \Omega_j^{-3}\sigma_{X_j}^2\sigma_{Y_j}^2\beta_{X_j}^2 + C_3 \sum_{j\in M_\ell} \Omega_j^{-4}\sigma_{X_j}^4\sigma_{X_j}^4$$

$$= \Theta(n^2\|\beta_X\|_4^4) + \Theta(n\|\beta_X\|_2^2) + \Theta(p),$$

and

$$\sum_{j\in M_\ell} E[\bar{B}_j^4] = C_4 \sum_{j\in M_\ell} \Omega_j^{-2}\beta_{X_j}^4 + C_5 \sum_{j\in M_\ell} \Omega_j^{-2}\sigma_{X_j}^2\beta_{X_j}^2 + C_6\theta_0^2 \sum_{j\in M_\ell} \Omega_j^{-3}\sigma_{X_j}^4\beta_{X_j}^2 + C_7\theta_0^2 \sum_{j\in M_\ell} \Omega_j^{-4}\sigma_{X_j}^4\sigma_{Y_j}^2\beta_{X_j}^2$$

$$+ C_8 \sum_{j\in M_\ell} \Omega_j^{-2}\sigma_{X_j}^4 + C_9\theta_0^2 \sum_{j\in M_\ell} \Omega_j^{-3}\sigma_{X_j}^6 + C_{10}\theta_0^4 \sum_{j\in M_\ell} \Omega_j^{-4}\sigma_{X_j}^4\sigma_{Y_j}^4$$

$$= \Theta(n^2\|\beta_X\|_4^4) + \Theta(n\|\beta_X\|_2^2) + \Theta(p).$$

Thus, by CS and the above,

$$\sum_{j\in M_\ell} E[\|\mu_j\|_2^3] \le \Big(\sum_{j\in M_\ell} E[\|\mu_j\|_2^4]\Big)^{\frac{1}{2}} \Big(\sum_{j\in M_\ell} E[\|\mu_j\|_2^2]\Big)^{\frac{1}{2}}$$

$$\le \big(O(n^2\|\beta_X\|_4^4) + O(n\|\beta_X\|_2^2) + O(p)\big)^{\frac{1}{2}} \big(O(n\|\beta_X\|_2^2) + O(p)\big)^{\frac{1}{2}}$$

$$= O(n^{\frac{3}{2}}\|\beta_X\|_4^2\|\beta_X\|_2) + O(\sqrt{p}n\|\beta_X\|_4^2) + O(n\|\beta_X\|_2^2) + O(p),$$

as required. □

Proof of Theorem 3 – Part II (Asymptotic distribution of the Focused estimator $\hat{\theta}$).

Let $\hat{\omega}_C$ denote a binary indicator that equals 1 only when S_0 is the AMSE minimising set of instruments, and let $\hat{\omega}_k$, $k \in [K]$ denote binary indicators which which equal 1 only when $S_0 \cup S_k$ is the AMSE minimising set of instruments. Then, $\hat{\omega}_C + \sum_{k=1}^K \hat{\omega}_k = 1$, and the Focused estimator can be written

$$\hat{\theta} - \theta_0 = \hat{\omega}_C(\hat{\theta}_{S_0} - \theta_0) + \sum_{k=1}^{K} \hat{\omega}_k(\hat{\theta}_{S_k} - \theta_0).$$

Let $U = (U_1, \dots, U_{2K+1})'$ be the normally distributed vector $U \sim N(0, \Delta)$. Then, under Assumptions 1-4, the asymptotic distribution of the Focused estimator is

$$\hat{\theta} - \theta_0 \overset{a}{\sim} \omega_C^\star U_1 + \sum_{k=1}^{K} \omega_k^\star \Big[U_{k+1} + \frac{b_{S_k}}{\eta_C + \eta_{S_k}}\Big],$$

as $n, p \to \infty$, where $\omega_C^\star = I\big\{\Delta_C \le \min_{k'\in[K]}\big([U_{K+k'+1} + (\eta_C + \eta_{S_{k'}})^{-2}b_{S_{k'}}]^2 - \Delta_B^{(k',k')} + \Delta_F^{(k',k')}\big)\big\}$ and $\omega_k^\star = (1-\omega_C^\star) \times I\big\{[U_{K+k+1} + (\eta_C + \eta_{S_k})^{-2}b_{S_k}]^2 - \Delta_B^{(k,k)} + \Delta_F^{(k,k)} = \min_{k'\in[K]}\big([U_{K+k'+1} + (\eta_C + \eta_{S_{k'}})^{-2}b_{S_{k'}}]^2 - \Delta_B^{(k',k')} + \Delta_F^{(k',k')}\big)\big\}$, $k \in [K]$.

To see this, note that for all $k \in [K]$, $\hat{\omega}_C$ and $\hat{\omega}_k$ are functions of the estimated AMSE which consist of consistent estimators of constants (see Lemma S.5) and $\hat{b}_{S_k}$. The result then follows by Slutsky's lemma and Part I which shows the joint convergence in distribution of $\hat{\theta}_C$, $\hat{b}_{S_k}$ and $\hat{\theta}_{S_k}$ over all instruments sets $S_0 \cup S_k$, $k \in [K]$. □

Proof of Theorem 4 (Worst case size distortion of Focused intervals).

Let $\hat{b} = \big(\hat{b}_{S_1}/(\hat{\eta}_C + \hat{\eta}_{S_1}), \ldots, \hat{b}_{S_K}/(\hat{\eta}_C + \hat{\eta}_{S_K})\big)'$ and $b = \big(b_{S_1}/(\eta_C + \eta_{S_1}), \ldots, b_{S_K}/(\eta_C + \eta_{S_K})\big)'$. Let $\mathcal{B}(b, \alpha_1) = \{b^\star : \Gamma(b, b^\star) \le \chi^2_K(\alpha_1)\}$ where $\chi^2_K(\alpha_1)$ denotes the $1-\alpha_1$ quantile of a random χ^2_K variable, $\Gamma(b, b^\star) = (b + M - b^\star)'\Delta_B^{-1}(b + M - b^\star)$, and $M \sim N(0_{K\times 1}, \Delta_B)$. Therefore, by Theorem 2, $\mathcal{B}(b, \alpha_1)$ is the limiting version of a $(1-\alpha_1) \times 100\%$ confidence region for the true asymptotic bias b.

Let $\mathcal{C} = (a_L(b'), a_U(b'))$ define a collection of $(1-\alpha_2) \times 100\%$ confidence intervals indexed by b', each constructed such that

$$P\big(a_L(b') \le \Lambda(b') \le a_U(b')\big) = 1 - \alpha_2 \quad \text{if} \quad b' = b, \quad \text{and}$$

$$P\big(a_L(b') \le \Lambda(b'') \le a_U(b')\big) \ge 1 - \alpha_2 - \gamma \text{ for all } b'' \in \mathcal{B}(b, \alpha_1).$$

Let $(a_L^\star, a_U^\star) = (a_L(b^\star), a_U(b^\star))$ be an interval such that $a_U(b^\star) - a_L(b^\star) \le a_U(b') - a_L(b')$ for all intervals $(a_L(b^\star), a_U(b^\star))$ and $(a_L(b'), a_U(b'))$ contained in $\mathcal{C}$, and $(b^\star, b') \in \mathcal{B}(b, \alpha_1)$.

Let $A = \{\Gamma(b, b) \le \chi^2_K(\alpha_1)\}$, so that A is the event that the limiting version of the confidence region for the asymptotic bias contains the true asymptotic bias b. Note that since $\Gamma(b, b^\star) \sim \chi^2_K$, we have $P(A) = 1 - \alpha_1$.

For every $b' \in \mathcal{B}(b, \alpha_1)$, we have $P\big(\{a_L^\star \le \Lambda(b') \le a_U^\star\} \cap A\big) + P\big(\{a_L^\star \le \Lambda(b') \le a_U^\star\} \cap A^c\big) \ge 1 - \alpha_2 - \gamma$ since $(a_L^\star, a_U^\star) \in \mathcal{C}$. Moreover, note that $P\big(\{a_L^\star \le \Lambda(b') \le a_U^\star\} \cap A^c\big) \le P(A^c) = \alpha_1$, and therefore, $P\big(\{a_L^\star \le \Lambda(b') \le a_U^\star\} \cap A\big) \ge 1 - \alpha_2 - \gamma - \alpha_1$ for all $b' \in \mathcal{B}(b, \alpha_1)$.

If A occurs, then by definition $b \in \mathcal{B}(b, \alpha_1)$. Hence, $P(\{a_L^\star \le \Lambda(b) \le a_U^\star\}) \ge 1 - \alpha_2 - \gamma - \alpha_1$. We can repeat these steps for different combinations of α_1 and α_2 such that $\alpha_1 + \alpha_2 = \alpha$ which leads to the result of the theorem. □

Additional simulation results. For the design described in Section 5.1, Figures S1–S3 show the results of the Focused estimator and interval for weaker instruments (the concentration parameter ranges from 10 to 50, compared with 40 to 200 in the main text). Figure S4 shows the results of confidence intervals when S_0 consists of invalid instruments, according to the design discussed in Section 5.5. We note that the patterns observed in Figures S1–S3 are very similar to those observed in Figures 2–4 in the main text, highlighting that for finite-sample performance, the relative strengths of the valid and additional instrument sets may be more important than their absolute strengths.

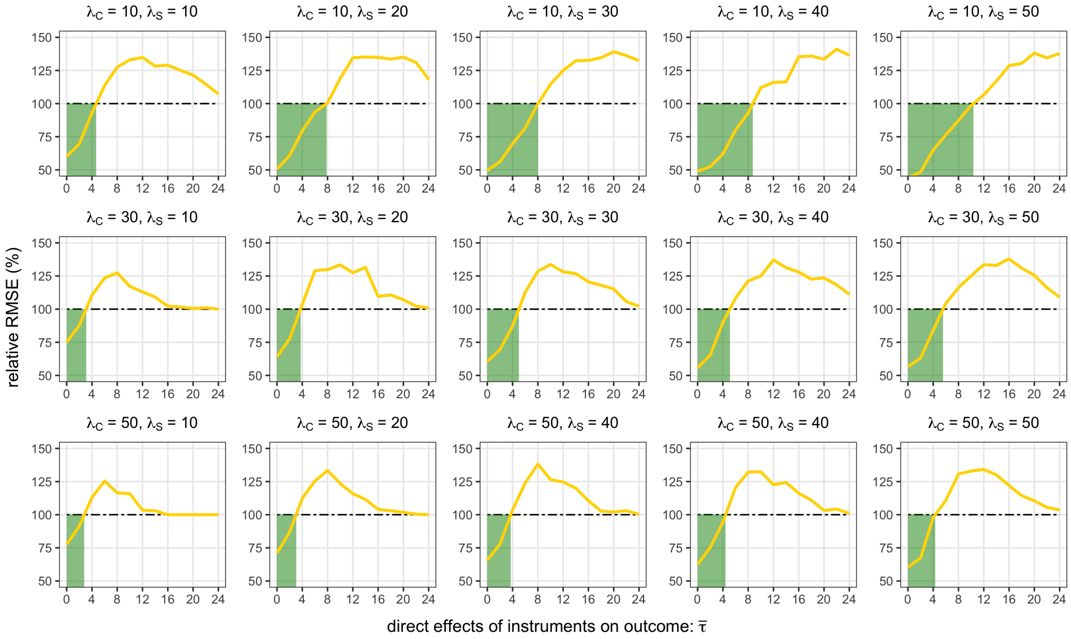

Figure S1. RMSE varying with the average instrument strength of S_0 (λ_C) and S (λ_S), and invalidness of S ($\bar{\tau}$).

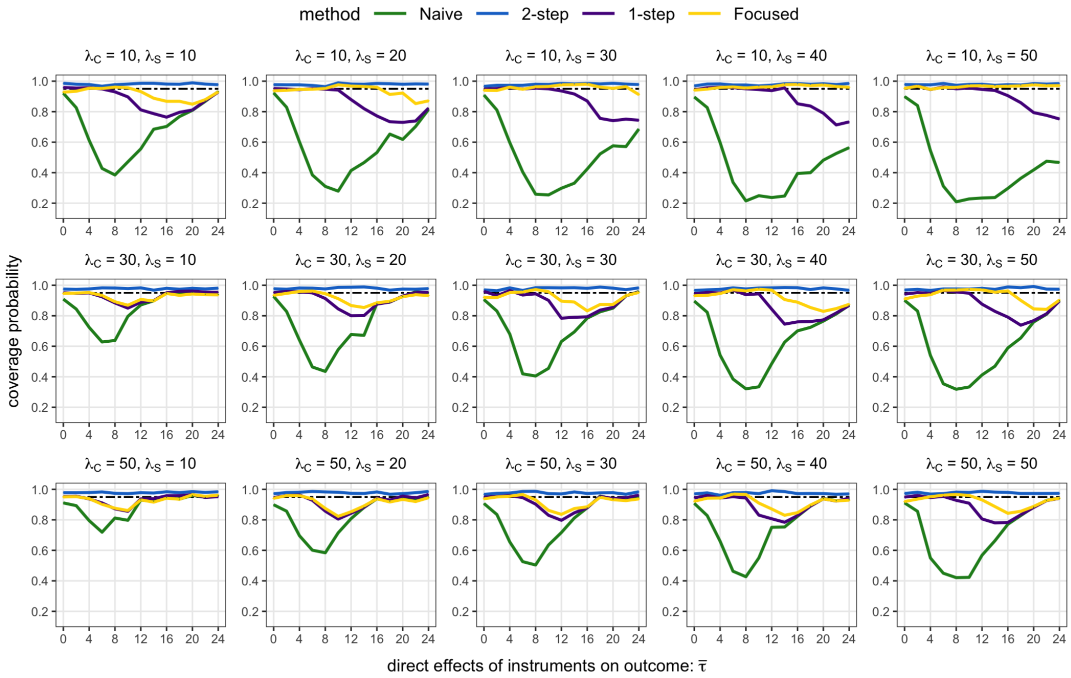

Figure S2. Coverage probabilities of confidence intervals (nominal coverage is $1 - \alpha = 0.95$).

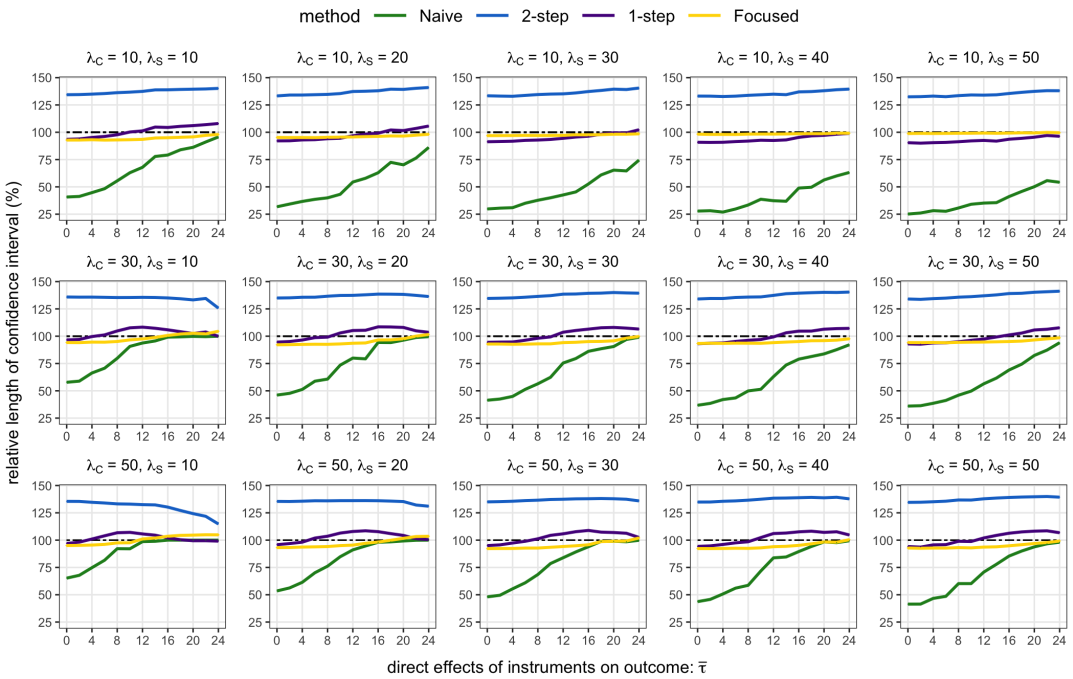

Figure S3. Length of confidence intervals relative to the Core interval (nominal coverage is $1 - \alpha = 0.95$).

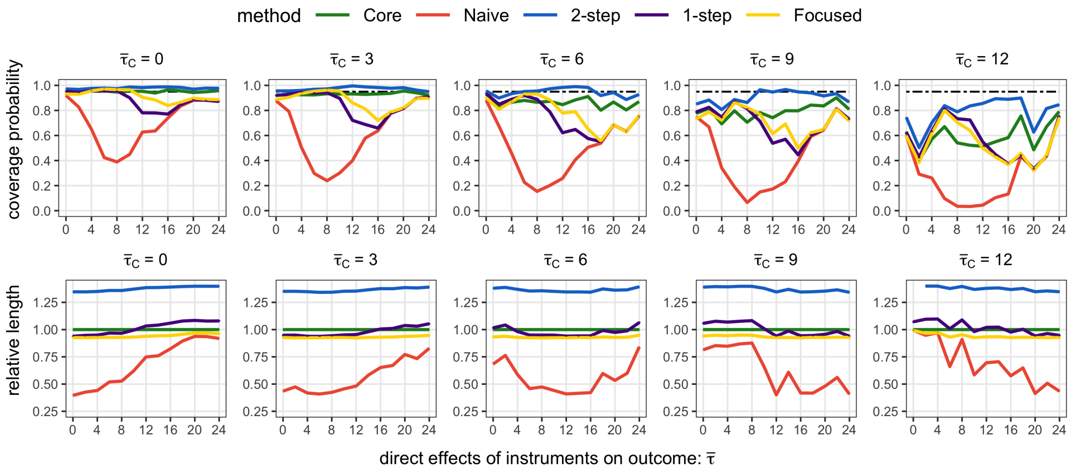

Figure S4. Coverage and length of 95% confidence intervals varying with invalidness of S_0 $(\bar{\tau}_C)$ and invalidness of S $(\bar{\tau})$.

Focused intervals when choosing the minimum feasible value of γ. In Figure 6 of the main text, we illustrated how the relative length of the Focused interval changed with the choice of γ. In particular, we found that the Focused interval was around 18% longer than the Core interval for the case where $\gamma = 0.05$ and all instruments were equally strong. To consider the performance of the Focused interval with lower values of γ, we ran simulation experiments that calculated the Focused interval at the minimum value of γ for which the interval was feasible.

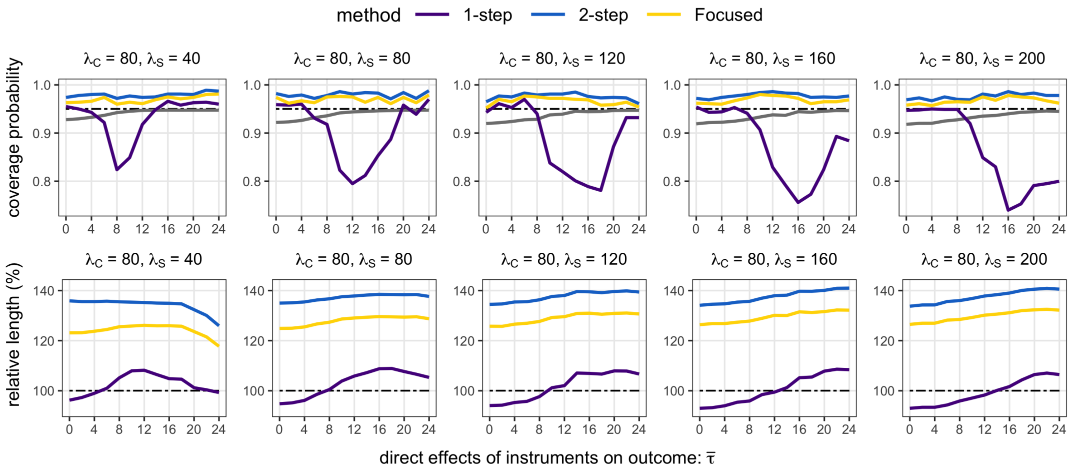

Figure S5. The dashed line in the first row is the nominal coverage $(1-\alpha=0.95)$. The Focused interval is computed for the lowest $\gamma=\gamma^\star$ for which the interval is feasible; the dashed line of nominal coverage minus the solid grey line equals $\gamma^\star$. The second row plots the length of confidence intervals relative to the Core interval.

Figure S5 plots simulations results under the same design discussed in Section 5.1 of the main text. The Focused interval with the minimum feasible γ (denoted $\gamma^\star$) over-covers, and the interval was generally over 20% longer in length than the Core interval. A higher threshold $\gamma^\star$ seems to be required for low values of direct effects $\bar{\tau}$; $\gamma^\star$ was as high as 0.032 when $\bar{\tau}=0$, whereas a maximum size distortion of only $\gamma^\star=0.005$ was required when $\bar{\tau}=24$. Instrument strengths λ_C and λ_S did not affect the required $\gamma^\star$ too much, although a slightly higher value of $\gamma^\star$ appeared to be needed as the additional instruments became stronger (i.e. for higher values of λ_S).

Optimal splits of α and γ. If an investigator wants to guarantee an asymptotic coverage probability of 0.95 from the Focused, we can consider any split of the nominal size α and worst case size distortion γ such that $\alpha+\gamma=0.05$. In particular, we can examine how the length of the Focused interval varies with splits of $(\alpha,\gamma)=(0.05-\gamma,\gamma)$ for $\gamma=0.125, 0.025, 0.375$. We calculated the optimal split among feasible values of $(\alpha,\gamma)=(0.05-\gamma,\gamma)$ that led to the shortest Focused interval with guaranteed $1-\alpha-\gamma=0.95$ coverage over all values of the direct effects $\bar{\tau}$.

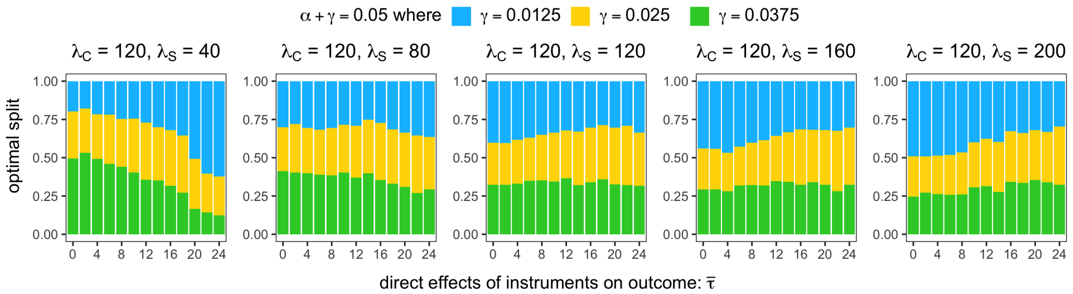

Figure S6. The optimal split of $(\alpha,\gamma)=(0.05-\gamma,\gamma)$ that leads to the shortest feasible Focused interval with guaranteed $1-\alpha-\gamma=0.95$ coverage probability over all values of the direct effects $\bar{\tau}$.

Figure S6 presents the optimal split of α and γ under the same simulation design discussed in Section 5.1. There appears to be no unanimously optimal choice among the splits we considered. When the core instruments were much stronger than the additional instruments $(\lambda_C=120, \lambda_S=40)$, then the optimal tolerance γ fell as $\bar{\tau}$ increased. Conversely, when the additional instruments were much stronger than the core instruments $(\lambda_C=120, \lambda_S=200)$, the optimal tolerance γ increased with $\bar{\tau}$.

The coverage probability of the Focused interval was over 0.95 for all values of $\bar{\tau}$. It is important to note that nominal coverage probability for the Focused interval was not $1-\alpha-\gamma=0.95$ in this case,

but rather $1-\alpha$. Therefore, the Focused interval was very conservative when compared to a 95% Core interval (between around 28% and 37% longer in length).

Pre-testing for bias. Theorem 2 of the main text can also be used to construct a pre-test of the potential bias that could result from the use of additional instruments. For a δ-level test of $H_0: b=0$ against the general alternative $H_1: b \neq 0$, we can simply compare the statistic $t_b = \hat{b}^2/\Delta_B$ against $q_1(\delta)$, the $(1-\delta)$-th quantile of a chi-squared random variable with 1 degree of freedom. If $t_b > q_1(\delta)$, then we reject the null hypothesis of $H_0: b=0$.

Another way to conduct a test of $H_0: b=0$ is to directly take the standardised difference of $\hat{\theta}_F$ and $\hat{\theta}_C$. Using Theorem 3 of the main text, we have that $\hat{\theta}_F - \hat{\theta}_C$ has mean zero under the null hypothesis $H_0: b=0$, and has variance $\Delta_F + \Delta_C - 2\Delta_E$. Thus, a Durbin-Wu-Hausman (DWH)-type test statistic (Hausman, 1978) is given by $t_b^\star = (\hat{\theta}_F - \hat{\theta}_C)^2/(\Delta_F + \Delta_C - 2\Delta_E)$. The statistic $t_b^\star$ is also asymptotically distributed as a chi-squared random variable with 1 degree of freedom as $n, p \to \infty$. Hence, we can reject the null hypothesis $H_0: b=0$ if $t_b^\star > q_1(\delta)$.

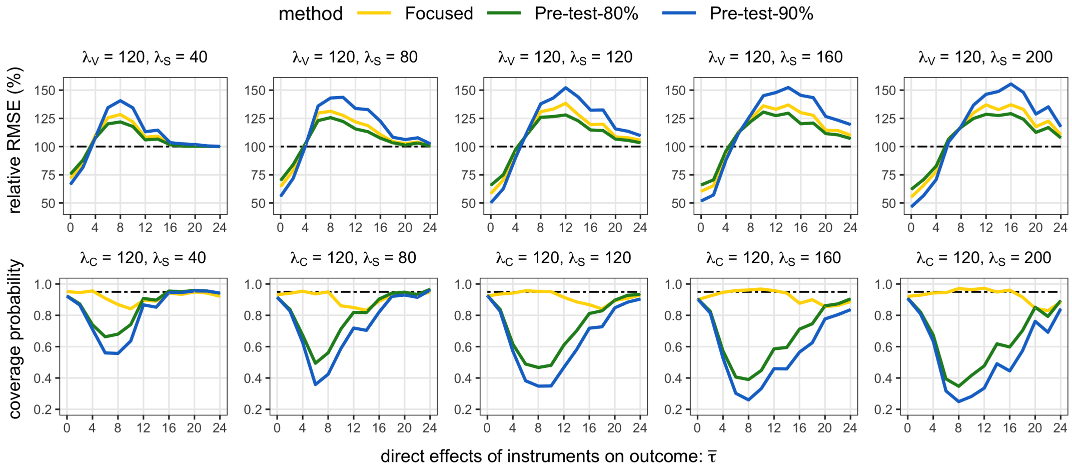

Figure S7. RMSE of the Focused estimator and an estimator based on pre-testing for bias relative to the Core estimator (first row). The second row plots the coverage probability of the Focused interval and confidence intervals based on bias pre-tests. The dashed line in the second row is the nominal coverage ($1-\alpha = 0.95$).

We could consider an instrument selection strategy based on conducting a pre-test of $H_0: b=0$. Such an approach first tests the null hypothesis $H_0: b=0$; if we cannot reject H_0, then we choose the full set of instruments since we have evidence that the full set of instruments does not lead to biased estimation. Otherwise, we stick with using the core instruments only. The focused instrument selection strategy has some parallels with this pre-testing approach: we can think of the focused selection as pre-testing the hypothesis $H_0: b=0$ with a fairly lenient threshold. With a more lenient threshold, we are more likely to include additional instruments even if they lead to slightly biased estimation.

To illustrate this idea, the top row of Figure S7 compares the estimation performance of the Focused estimator with estimators based on pre-testing for bias using significance thresholds of $\delta = 0.2$ (Pre-test-80%) and $\delta = 0.1$ (Pre-test-90%). For this particular simulation design (which is as described in Section 5.1 of the main text), it appears that somewhere between a $\delta = 0.1$ and $\delta = 0.2$ pre-testing significance threshold could lead to very similar estimation performance to the Focused estimator. However, more generally, it is not clear what a suitable choice of δ should be. On the other hand, the focused instrument selection method offers a principled way to minimise estimation risk that does not require investigators to choose δ.

Further, choosing instruments based on pre-testing for bias can lead to very poor inference if the uncertainty in instrument selection is not taken into account. The second row of Figure S7 highlights the poor coverage performance of naive confidence intervals that do not account for pre-test uncertainty.

For the Focused interval, the choice of the worst-case size distortion γ for the Focused interval was set to $\gamma = 0.2$.

Power for testing $H_0 : \theta_0 = 0$. A nominal α-level test of the null hypothesis $H_0 : \theta_0 = 0$ can be conducted by checking whether or not 0 is contained in a $(1-\alpha) \times 100\%$ Focused confidence interval $CI_F(\alpha, \gamma)$; if the Focused interval does not contain 0, then we reject the null hypothesis.

This would be a consistent test under fixed alternatives $H_1 : \theta_0 = \theta_\star$, for any constant $\theta_\star \neq 0$. To see this, note that the Focused interval is given by $CI_F(\alpha, \gamma) = [\hat{\theta} - q_2^\star(\alpha, \gamma), \hat{\theta} - q_1^\star(\alpha, \gamma)]$, where $q_1^\star(\alpha, \gamma)$, $q_2^\star(\alpha, \gamma)$ are certain quantiles of the distribution of $\Lambda(b^\star(\alpha, \gamma))$, and where $\Lambda(b^\star(\alpha, \gamma)) = O_P(1/\sqrt{n}\|\beta_X\|_2)$ as $n, p \to \infty$. Hence, also noting that $\hat{\theta}$ consistently estimates $\theta_\star$ under H_1 by Lemma S.1 of Supplementary Material, we have that $P(\theta_0 \in CI_F(\alpha, \gamma)) \to 0$ as $n, p \to \infty$.

To compare the power performance of the different inference procedures discussed in Section 4.2, we consider simulation experiments as described in Section 5.1 of the main text but under local alternatives. Figure S8 plots power curves under local alternatives $H_1 : \theta_0 = \kappa/\sqrt{n}$, for varying values of κ shown on the x-axis. On the y-axis, we plot the rejection frequencies of tests that reject the null hypothesis $H_0 : \theta_0 = 0$ if 0 is not contained in a nominal $(1-\alpha) \times 100\%$ confidence interval.

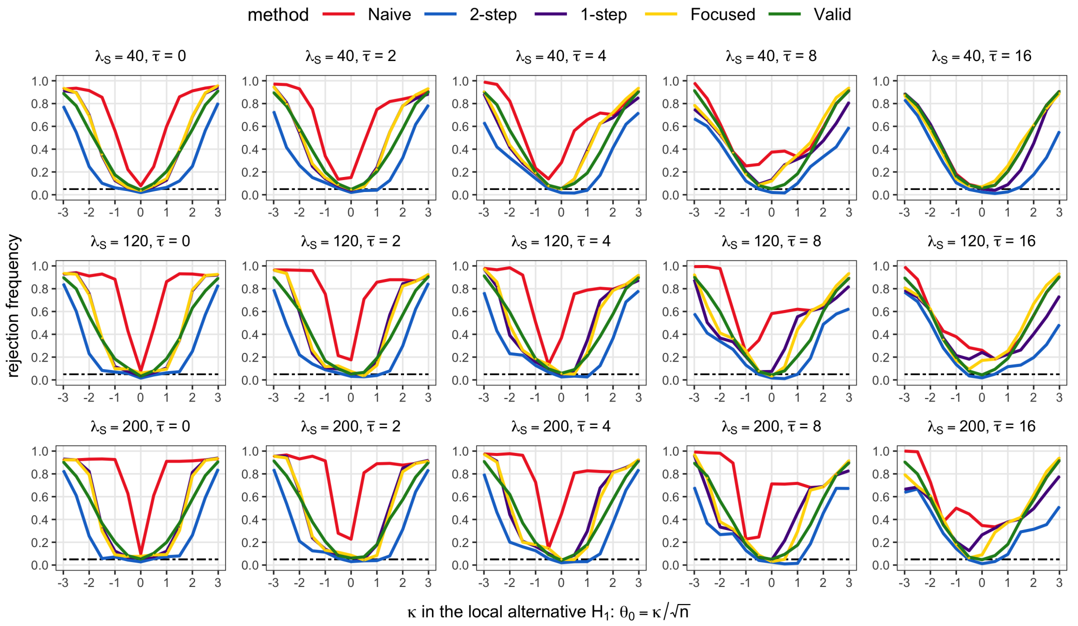

Figure S8. Power for testing the null $H_0 : \theta_0 = 0$ under the local alternative $H_1 : \theta_0 = \kappa/\sqrt{n}$ for varying κ. The dashed line is the nominal size of the tests ($\alpha = 0.05$). The worst case size distortion of the Focused intervals were set at $\gamma = 0.2$. The concentration parameter for the core instruments was $\lambda_C = 120$.

From Figure S8, the size performance of the Focused inference procedure is mostly consistent with the coverage performance of Focused intervals shown in Figure 4 in Section 5.3 of the main text. If the additional instruments are valid or slightly invalid ($\bar{\tau} \leq 4$), then Focused inference controls type I error rates close to the nominal 5% level. As the additional instruments become more invalid, then there is type I error inflation (the rejection frequency under $\kappa = 0$ is around 18% when $\bar{\tau} = 16$ and all instruments are equally strong, $\lambda_C = \lambda_S = 120$), but it is less than the nominated worst case size distortion of $\gamma = 0.2$.

In terms of power, the Focused inference approach appears to be more conservative than standard inference based on the Core intervals for subtle deviations away from the null (over the range $|\kappa| < 1.5$ when $\bar{\tau} \leq 2$). Under larger departures from the null, Focused inference tends to be more powerful. For example, for the case where all instruments are equally strong ($\lambda_C = \lambda_S = 120$) and $\bar{\tau} = 4$, Focused

inference controls type I error rates at the nominal 5% level, while the rejection frequency of the Focused inference procedure is around 20% higher than Core inference when $\kappa = 2$.

We also note that the performance of the 2-step and Naive inference procedures are also consistent with their coverage and length results presented in Section 5.3 of the main text. In particular, the Naive inference procedure can be severely over-sized, with type I error rates of 70% when $\lambda_S = 200$ and $\bar{\tau} = 8$, whereas 2-step inference controls type I error rates across the range of λ_S and $\bar{\tau}$ values considered, but the latter approach is very conservative.

REFERENCES

BENTKUS, V. (2005). A Lyapunov-type bound in R^d. *Theory of Probability and Its Applications* **49** 400–410.

HAUSMAN, J. A. (1978). Specification tests in econometrics. *Econometrica* **46** 1251–1271.

ZHAO, Q., WANG, J., HEMANI, G., BOWDEN, J. and SMALL, D. S. (2020). Statistical inference in two-sample summary-data Mendelian randomization using robust adjusted profile score. *Annals of Statistics* **48** 1742 – 1769.
